# Mathematical Analysis and Experimental Implementation of a Self-Calibration Method for Ultra-Precision Displacement Sensors

**DOI:** 10.3390/s26206541

**Published:** 2026-10-15

**Authors:** Zhiyang Zhang, Xiaoyang Liu, Ryo Sato, Wei Gao

**Affiliations:** Department of Finemechanics, Tohoku University, 6-6-01 Aramaki-Aza Aoba, Aoba-ku, Sendai 980-8579, Miyagi, Japan; zhang.zhiyang.s2@dc.tohoku.ac.jp (Z.Z.); liu.xiaoyang.s4@dc.tohoku.ac.jp (X.L.); i.ko.c2@tohoku.ac.jp (W.G.)

**Keywords:** displacement sensor, linearity error, mathematical analysis, self-calibration, artificial neural network

## Abstract

**Highlights:**

**What are the main findings?**

- Thermal drifts in sensor outputs during the sampling process are the primary source of low-frequency components in self-calibration errors.
- Fitting errors of the discrete calibration data are the primary source causing high frequency components of self-calibration errors.

**What are the implications of the main findings?**

- Shortening the calibration time through an automated self-calibration setup is an effective way to reduce self-calibration errors caused by thermal drifts in sensor outputs.
- Artificial neural network (ANN)-based data fitting is an effective way to reduce self-calibration errors associated with conventional least-squares polynomial fitting.

**Abstract:**

This paper starts from a revisit to the self-calibration concept of displacement sensors proposed previously by Kiyono et al. In this self-calibration scheme, two displacement sensors are used to perform a mutual calibration operation to determine their linearity error components without using a higher-accuracy reference, while their sensitivity coefficients are assumed to be known and are determined separately. A set of mathematical equations are newly established in this paper to provide a mathematical basis for the self-calibration concept. Based on the established mathematical equations, the parameters influencing the calibration results are investigated through mathematical analysis and simulation. As new findings of this paper, it is verified that the polynomial fitting errors of the discrete calibration data and the thermal drifts of the sensor outputs during the sampling process have significant impacts on the calibration results and must be carefully suppressed in practical operations. Based on these findings, a calibration system is designed and constructed for calibration experiments in which motor-driven stages are employed to shorten the calibration time and to reduce the influences of thermal drifts. The experimental results are compared with those obtained using a laser interferometer, from which the effectiveness of the constructed calibration system is verified. An Artificial Neural Network (ANN) is also introduced in this paper for fitting the calibration data containing high-frequency components instead of the conventional least-squares polynomial fitting method, which had been employed in the previous work. The self-calibration results show better repeatability than the interferometer-based calibration results. The uncertainty associated with the proposed self-calibration method was preliminarily evaluated using a Monte Carlo analysis based on experimental data.

## 1. Introduction

In the field of ultra-precision machining and measurement, high-accuracy displacement sensors play a decisive role in surface metrology [1,2,3,4,5,6]. Calibration is an essential process to obtain a calibration function of the sensor before a displacement sensor can be used [7]. In such a calibration function, the input displacement is taken as the horizontal coordinate, and the output of the sensor is taken as the vertical coordinate [8]. The linearity errors of a displacement sensor are defined as the deviations of the calibration curve from a specified reference line, often a best-fit straight line of the curve [9]. The deviations stem from complex physical phenomena inherent to the sensing technology and manufacturing conditions [10,11,12,13,14]. In the specification sheet of a commercially available displacement sensor, the maximum value of the linearity errors is typically expressed as a percentage of the sensor full measurement range [15]. Such a value in the specification sheet provides a simple and clear indication of the linearity of the sensor output.

On the other hand, the linearity errors across the sensor measurement range introduce measurement errors in precision positioning and surface metrology [16,17]. In precision positioning, the linearity errors of a displacement sensor degrade the positioning accuracy of a stage, propagating through the control system to manifest as geometric and form errors on the final machined workpiece [18,19,20,21]. In surface metrology, the linearity errors directly introduce error components in the measured results of surface form and surface finish [22,23]. On the other hand, most ultra-precision displacement sensors have high stability, meaning that the calibration function including the linearity errors is repeatable. This means that linearity errors of the sensor can be compensated based on the calibration function. As a result, the measurement errors of the sensor can be reduced. This is especially important and effective when an ultra-precision displacement sensor is employed for measurement and manufacturing processes with nanometric precision [24,25].

As described above, the linearity errors of an ultra-precision displacement sensor can be obtained from the calibration function of the sensor. In an existing calibration system for getting the calibration function, the displacement sensor is calibrated by a working length standard, such as a calibrated laser interferometer [7,26,27,28]. In such a system, the calibrated sensor is linked to the definition of the meter and the national length standard through a traceability chain including different levels of length standards, from the primary length standard in a national metrology institute, the secondary length standards and the reference length standards in accredited calibration laboratories and working standards in manufacturing facilities [29,30,31,32]. Within this calibration framework, the working length standard used to calibrate the displacement sensor must be periodically sent to an accredited calibration laboratory for calibration every one or two years [33]. The displacement sensor can also be directly sent to an accredited calibration laboratory for calibration without using a working length standard. In either case, the accredited calibration laboratory issues a calibration certificate upon completion, to demonstrate the traceability of the calibration results.

The calibration function and the associated linearity errors obtained in the existing calibration system have the advantage of high reliability because they are traceable to the national length standard [7]. This is extremely important for applications where absolute accuracy of the measured displacement is required. However, there are two major problems with the existing calibration system. Firstly, the calibration cost is high. For example, a calibrated laser interferometer used as the working length standard is often much more expensive than the displacement sensor for calibration. It is often unavailable in many manufacturing facilities and university laboratories [34]. Secondly, the calibration accuracy is limited by that of the working length standard. This is particularly a problem for the calibration of an ultra-precision displacement sensor, which can have better performance than the working length standard in terms of measurement resolution and stability.

Meanwhile, self-calibration techniques have long been used in precision engineering for accurate measurements without using any high-accuracy reference standard [8,20,35]. In 1993, Kiyono et al. [36] proposed a self-calibration concept for the linearity errors of a pair of displacement sensors, in which two identical displacement sensors were mutually calibrated without relying on a higher-accuracy reference standard where a conventional least-squares polynomial fitting method was employed to fit the discrete calibration data. However, although the concept was clearly explained with some initial experimental data, mathematical equations of the calibration principle have not been established in detail, which is essential as the mathematical foundation of the calibration method. Mathematical analysis has also not been carried out to investigate the influence of various parameters, such as magnification ratio, number of sampling points, sensor sensitivity, and the effect of sensors’ off-axis motions (caused by thermal expansion or backlash).

As subsequent research of the self-calibration concept by Kiyono, this paper establishes a set of mathematical equations for analysis of the self-calibration process in Section 2, based on which the effects of the influencing factors on the self-calibration results are investigated in Section 3. In Section 4, an automated self-calibration system is designed and constructed for demonstrating the feasibility of the self-calibration method. In response to the high-frequency components in the calibration results, an Artificial Neural Network (ANN) [37,38,39,40,41,42,43,44,45,46,47,48,49] is employed to establish a model for fitting the discrete calibration data instead of the conventional least-squares polynomial fitting method, which had been employed in the previous work by Kiyono [36].

## 2. Establishment of Mathematical Fundamentals for the Self-Calibration Method Using a Pair of Displacement Sensors

For an ideal displacement sensor, namely Sensor A, the input displacement z and the corresponding sensor output *v* have a linear relationship over the full range from 0 to *z*_F_ which can be expressed by a simple linear function as follows:

*v*_A_(*z*) = *k*_A_*z*,   (0 ≤ *z* < *z*_F_) (1)

where *k*_A_ is referred to as the sensitivity coefficient of Sensor A. The output *v* is assumed to be zero when *z* = 0 for simplicity. For an actual displacement sensor, however, the input displacement and the corresponding sensor output will have a nonlinear relationship, which can be expressed as follows:

*v*_A_(*z*) = *k*_A_*z* + *e*_A_(*z*),   (0 ≤ *z* < z_F_) (2)

where *e*_A_(*z*) is the deviation of the sensor output from the ideal linear function shown in Equation (1). Here, Equation (2) is referred to as the calibration function. *e*_A_(*z*) is referred to as the linearity error of Sensor A. Note that *e*_A_(*z*) is a small fraction of the sensor measurement range. It should also be noted that the calibration function is a continuous function that can provide the relationship for any values of the input and output of the sensor.

**Figure 1:**
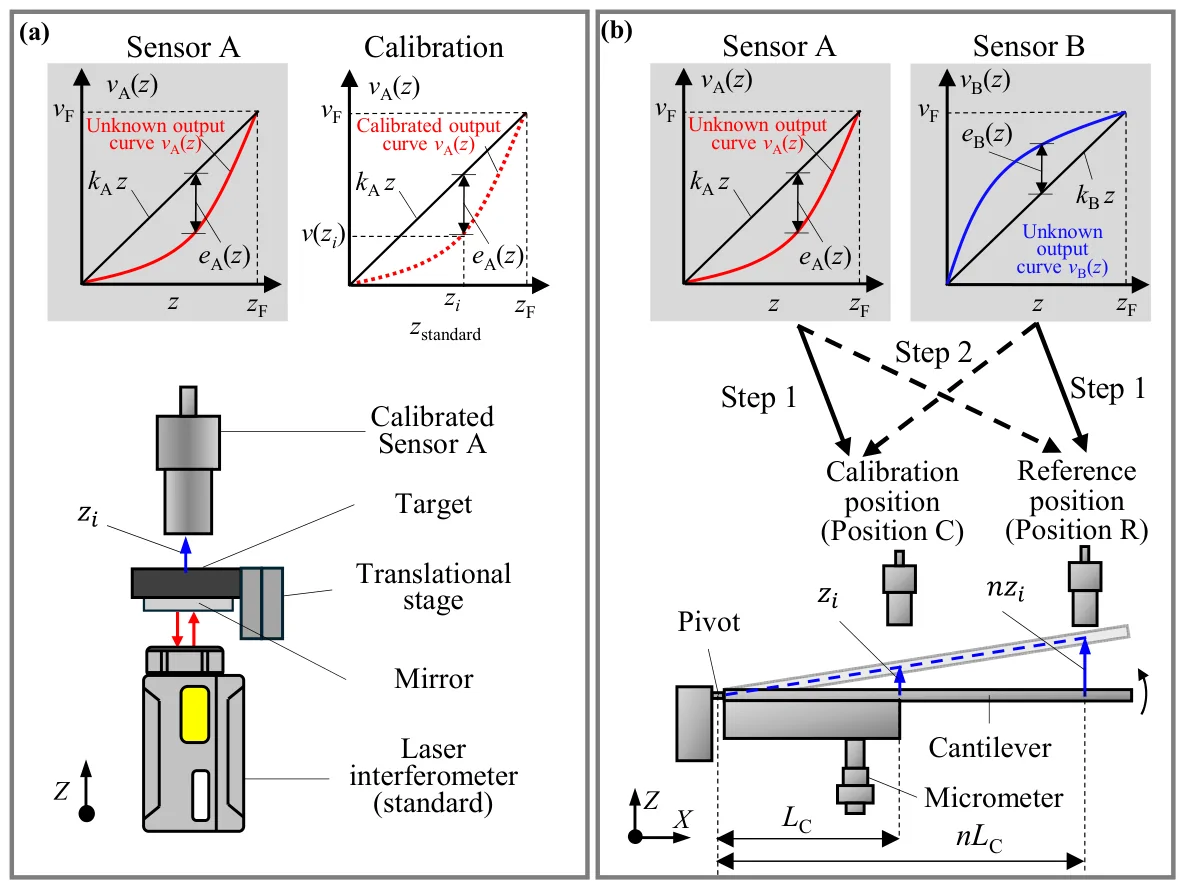
From the conventional calibration concept using a standard sensor to the self-calibration concept using a pair of two uncalibrated sensors with unknown linearity error components *e*_A_(*z*) and *e*_B_(*z*). (**a**) Conventional calibration concept. (**b**) Self-calibration concept by Kiyono [36].

Calibration is an essential process for obtaining the calibration function shown in Equation (2), which is important for accurate use of the sensor. Figure 1a shows a conventional calibration setup where an accurate displacement sensor, often a length interferometer, is employed as the standard of calibration. A target is moved by a translational stage across the measurement range of the displacement sensor. A series of discrete positions of the target along the moving axis of the stage are sampled by the two sensors simultaneously. Least-squares polynomial fitting is then carried out against the obtained discrete data (*v*_A_(*z_i_*), *z_i_*) to obtain a continuous calibration function shown in Equation (2). Both the sensitivity component *k*_A_ and the linearity error component *e*_A_(*z*) can be obtained from the calibration results, which is one of the main advantages of this method. However, as stated in the Introduction section, the conventional calibration method has the disadvantage of high cost. It is also impossible to calibrate a displacement sensor that has a comparable or higher performance than the standard sensor, which is a significant problem in calibration of ultra-precision displacement sensors.

On the other hand, compared with the linearity error component *e*_A_(*z*), it is much easier to obtain the sensitivity component *k*_A_, which can be made by using a gauge block with a length that is equal to the measurement range of the displacement sensor. Based on this fact, Kiyono et al. proposed a self-calibration method for *e*_A_(*z*) without using an accurate standard sensor, assuming that the displacement sensor has a known *k*_A_ [36]. As can be seen in Figure 1b, another displacement sensor (Sensor B), whose calibration function is shown below, is added for the self-calibration.

*v*_B_(*z*) = *k*_B_*z* + *e*_B_(*z*),   (0 ≤ *z* < *z*_F_) (3)

where *k*_B_ is a known sensitivity component, and *e*_B_(*z*) is an unknown linearity error component. In the present method, the sensitivity coefficients *k*_A_ and *k*_B_ are assumed to be known and are not estimated by self-calibration. The self-calibration method is used specifically to determine the linearity error components *e*_A_(*z*) and *e*_B_(*z*). The complete calibration functions can subsequently be reconstructed by combining the independently determined sensitivity coefficients with the self-calibrated linearity-error components. Therefore, the term “self-calibration” in this paper refers to the calibration of the linearity-error components rather than to a reference-free determination of the absolute sensor scale.

**Figure 2:**
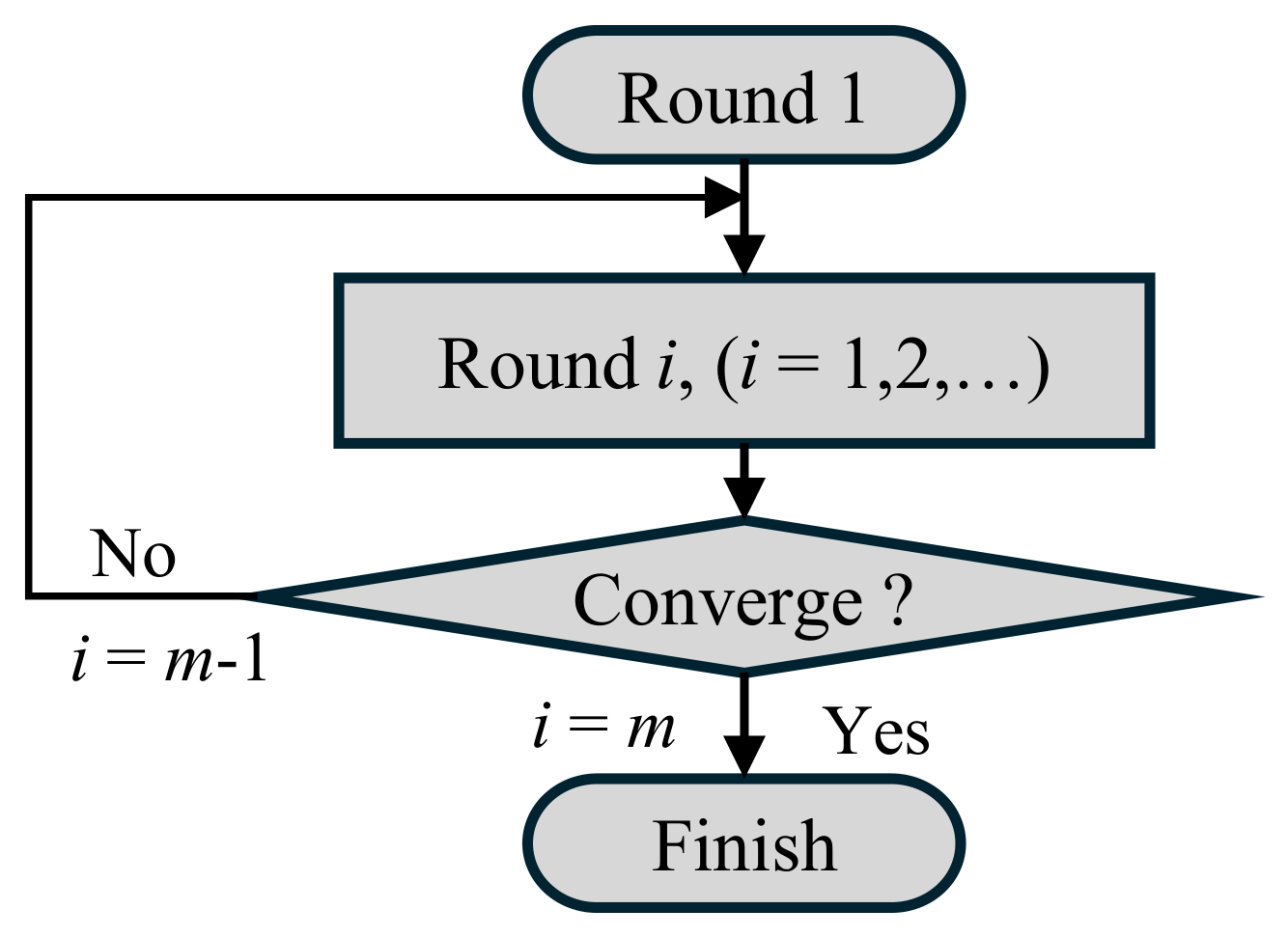
Iteration logic of the self-calibration rounds.

As shown in Figure 1b, two different positions C and R on one side of a cantilever are set as the calibration position and the reference position, respectively. For the sake of clarity, the two sensors are assumed to have an identical measurement range *z*_F_ and an identical full output *v*_F_. The distances of Positions C and R from the pivot of the cantilever are denoted by *L*_C_ and *nL*_C_, respectively, where *n* is referred to as the magnification ratio. The magnification ratio *n* is a positive real number larger than 1 and is not required to be an integer. The cantilever can be moved about its pivot by a translational stage set on the other side of the cantilever. The self-calibration is typically repeated for several rounds, as shown in Figure 2. In each calibration round, the self-calibration operations are carried out in the two sensor setups shown in Figure 1b, where *n* = 2 is used only in the figure for clarity.

(1) Self-calibration at Round 1

As can be seen in Figure 2, the self-calibration starts from Round 1 with totally unknown linearity errors *e*_A_(*z*) and *e*_B_(*z*) of the two sensors. The calibration operations in Round 1 are carried out in the two sensor setups.

Setup 1: Calibrating Sensor A with Sensor B

**Figure 3:**
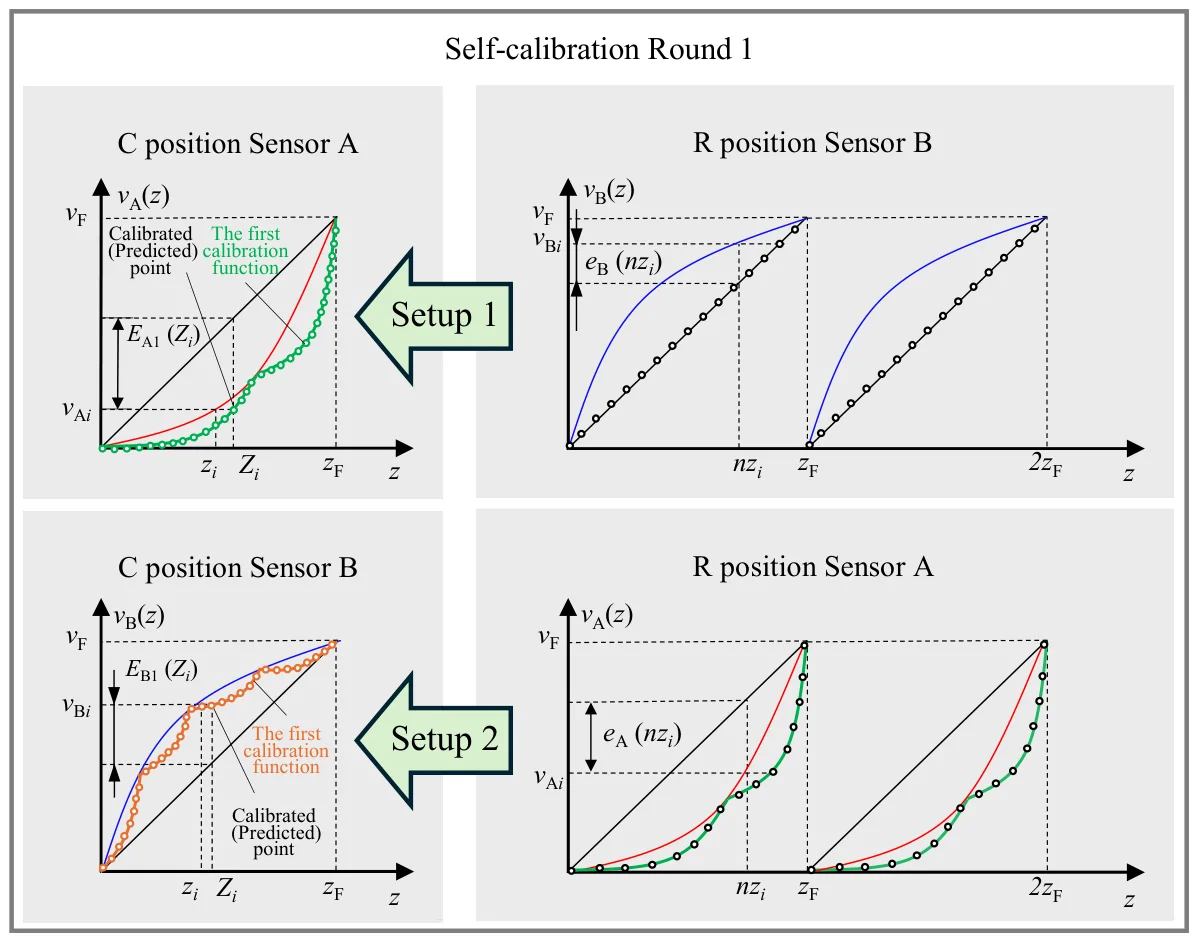
Self-calibration process of Round 1.

In this setup, Sensors A and B are located at Positions C and R, respectively, to obtain the first calibration result of Sensor A shown in Figure 3. This can be understood that the linearity error of Sensor A is calibrated by Sensor B. It should be noted that at the beginning of calibration, both the linearity errors of Sensors A and B are completely unknown. At first, the cantilever is moved by the translational stage so that the output of Sensor A is sampled across the first calibration range from 0 to *v*_F_/*n* while that of Sensor B is sampled across its full range *v*_F_. Sensor B is then moved by the translational stage until its output is returned to zero, allowing Sensor A to be calibrated over the next calibration range. For a non-integer *n*, this operation provides ⌊*n*⌋ complete calibration ranges and one final partial calibration range. The total number of calibration ranges is therefore ⌈*n*⌉. Here, ⌊*n*⌋ denotes the greatest integer not larger than *n*, while ⌈*n*⌉ denotes the smallest integer not smaller than *n*.

Consider the first calibration range from 0 to *z*_F_/*n*. Let *N* denote the total number of paired samples acquired over the full calibration range from 0 to *z*_F_, and let *i* = 1,…,*N* denote the sample index. For a sample *i* within the first calibration range, the output *v*_A_(*z_i_*) of Sensor A at Position C is denoted by *v*_A*i*_, where *z_i_* is the input displacement. The corresponding input displacement at Position R is *nz_i_*, and the output *v*_B_(*nz_i_*) of Sensor B is denoted by *v*_B*i*_. Both *z_i_* and *nz_i_* are unknown. A predicted input displacement *Z_i_* for the first calibration function can be obtained from *v*_B*i*_ as follows:

(4) $$Z_{i}=\frac{v_{Bi}}{nk_{B}}=\frac{k_{B}{\mathrm{nz}}_{i}+e_{B}({\mathrm{nz}}_{i})}{nk_{B}}=z_{i}+\frac{e_{B}(nz_{i})}{nk_{B}},\;\;\;(0\leq z_{i}<\frac{z_{F}}{n})$$

Substituting *v*_A*i*_ and *Z_i_* into Equation (2) gives:

(5) $$v_{Ai}=k_{A}Z_{i}+E_{A1,1}\left(Z_{i}\right),\;\;\;(0\leq z_{i}<\frac{z_{F}}{n})$$

where *E*_A1,1_ is the first predicted linearity error component over the first calibration range from 0 to *v*_F_/*n*. Note that *E*_A1,1_(*z_i_*) is assumed to be equal to *E*_A1,1_(*Z_i_*) since the difference between *z_i_* and *Z_i_* only has a secondary effect on *E*_A1,1_. Substituting Equation (4) into Equation (5) gives:

(6) $$v_{Ai}=k_{A}z_{i}+\frac{k_{A}}{k_{B}}\frac{e_{B}(nz_{i})}{n}+E_{A1,1}(z_{i}),\;\;\;(0\leq z_{i}<\frac{z_{F}}{n})$$

Substituting Equation (2) into the left-hand side of Equation (6) gives:

(7) $$k_{A}z_{i}+e_{A}(z_{i})=k_{A}z_{i}+\frac{k_{A}}{k_{B}}\frac{e_{B}(nz_{i})}{n}+E_{A1,1}(z_{i}),\;\;\;(0\leq z_{i}<\frac{z_{F}}{n})$$

*E*_A1,1_(*z_i_*) can then be evaluated as follows:

(8) $$E_{A1,1}\left(z_{i}\right)=e_{A}\left(z_{i}\right)-{\Delta e}_{A1,1}\left(z_{i}\right),\;\;\;(0\leq z_{i}<\frac{z_{F}}{n})$$

(9) $${\Delta e}_{A1,1}\left(z_{i}\right)=\frac{k_{A}}{k_{B}}\frac{e_{B}(nz_{i})}{n},\;\;\;(0\leq z_{i}<\frac{z_{F}}{n})$$

where Δ*e*_A1,1_(*z_i_*) is the calibration error of *E*_A1_(*z_i_*) over the first calibration range from 0 to *v*_F_/*n*. If *k*_A_ = *k*_B_, Equation (9) becomes

(10) $${\Delta e}_{A1,1}\left(z_{i}\right)=\frac{e_{B}(nz_{i})}{n},\;\;\;(0\leq z_{i}<\frac{z_{F}}{n})$$

The linearity error component *e*_A1,1_(*z_i_*) of Sensor A can be predicted by *E*_A1,1_(*z_i_*) with an error of *e*_B_(*nz_i_*)/*n* although *e*_B_ is still unknown. The same calibration is then carried out over the subsequent calibration ranges so that Sensor A can be calibrated over its full range from 0 to *z*_F_. Equation (5) can then be extended to as follows:

(11) $$v_{Ai}=k_{A}Z_{i}+\sum_{j=0}^{\left\lceil n\right\rceil -1}E_{A1,\;j+1}\left(z_{i}-jz_{F}/n\right),\;\;i=1,\ldots ,N,\;\;\;\left(0\leq z_{i}\leq z_{F}\right)$$

In Equation (11), the *j*th shifted component is used only over its corresponding calibration range. The terms with *j* = 0,…,⌊*n*⌋ − 1 correspond to the complete calibration ranges, while the term with *j* = ⌊*n*⌋ corresponds to the final partial calibration range.

Then a least-squares polynomial fitting is made against the discrete calibration data set of *v*_A*i*_ and *Z_i_* over the full calibration range of 0 to *z*_F_ for obtaining the 1st predicted calibration function of Sensor A over the full calibration range, which is a continuous function, as follows:

(12) $$v_{A1}\left(z\right)=k_{A}z+\sum_{j=0}^{\left\lceil n\right\rceil -1}E_{A1,j+1}\left(z-jz_{F}/n\right)+\delta_{A1}\left(z\right),\;\;\;\left(0\leq z\leq z_{F}\right)$$

where *δ*_A1_ is an error component arising in the fitting process of the discrete calibration data set for getting the 1st predicted calibration function of Sensor A. Each shifted component in Equation (12) is defined only over the corresponding complete or partial calibration range specified above. Rewriting Equation (12) gives the 1st predicted calibration function over the first calibration range of 0 to *z*_F_/*n*:

*v*_A1_(*z*) = *k*_A_*z* + *E*_A1,1_(*z*) + *δ*_A1_(*z*),   (0 ≤ *z* < *z*_F_/*n*) (13)

The calibration error shown in Equation (9) can also be rewritten as the following continuous functions over the first calibration range from 0 to *z*_F_/*n*, and the full calibration range from 0 to *z*_F_, respectively.

(14) $${\Delta e}_{A1,1}\left(z\right)=\frac{k_{A}}{k_{B}}\frac{e_{B}(\mathrm{nz})}{n}-\delta_{A1}\left(z\right),\;\;\;(0\leq z<z_{F}/n)$$

(15) $${\Delta e}_{A1}\left(z\right)=\sum_{j=0}^{\left\lceil n\right\rceil -1}\frac{k_{A}}{k_{B}}\frac{e_{B}[n(z-jz_{F}/n)]}{n}{-\delta }_{A1}\left(z\right),\;\;\;\left(0\leq z\leq z_{F}\right)$$

Setup 2: Calibrating Sensor B with Sensor A

In this setup, Sensors B and A are placed at Positions C and R, respectively, for calibrating Sensor B with Sensor A as shown in Setup 2 of Figure 3. Note that Sensor A has a 1st predicted calibration function shown in Equations (12) or (13). The calibration operations are the same as those in Setup 1. Similarly, the 1st predicted calibration function of Sensor B over the first subinterval of first calibration range of 0 to *z*_F_/*n*^2^ can be obtained as follows:

*v*_B1_(*z*) = *k*_B_*z* + *E*_B1,1_(*z*) + *δ*_B1_(*z*),   0 ≤ *z* < *z*_F_/*n*^2^ (16)

Note that the first subinterval of first calibration range of Sensor B is from 0 to *z*_F_/*n*^2^, which is 1/*n* of that in Setup 1. The input displacement to Sensor B is predicted by using the output of Sensor A shown in Equation (13), which is over the range of 0 to *z*_F_/*n*. Similarly, the 1st predicted calibration function of Sensor B over the full calibration range of 0 to *z*_F_ can be written by:

(17) $$v_{B1}\left(z\right)=k_{B}z+\sum_{j=0}^{\left\lceil n\right\rceil -1}E_{B1,j+1}\left(z-jz_{F}/n\right)+\delta_{B1}\left(z\right),\;\;\;(0\leq z\leq z_{F})$$

where *δ*_B1_ is an error component arising in the fitting process of the discrete data for getting the 1st predicted calibration function of Sensor B. *E*_B1,1_ is the first predicted linearity error component of Sensor B over its first subinterval from 0 to *z*_F_/*n*^2^.

The corresponding calibration errors of Sensor B over its first subinterval of first calibration range of 0 to *z*_F_/*n*^2^ and the full calibration range of 0 to *z*_F_ can be expressed in the following equations, respectively.

(18) $${\Delta e}_{B1,1}\left(z\right)=\frac{e_{B}\left(n^{2}z\right)}{n^{2}}-\frac{k_{B}}{k_{A}}\frac{\delta_{A1}(\mathrm{nz})}{n}-\delta_{B1}\left(z\right),\;\;\;(0\leq z<\frac{z_{F}}{n^{2}})$$

(19) $${\Delta e}_{B1}\left(z\right)=e_{B}\left(z\right)-\sum_{j=0}^{\left\lceil n\right\rceil -1}E_{B1,j+1}\left(z-jz_{F}/n\right)-\delta_{B1}\left(z\right)\;\begin{array}{c} =\sum_{j=0}^{\left\lceil n\right\rceil -1}\left[\frac{1}{n^{2}}\sum_{l=0}^{\left\lceil n\right\rceil -1}e_{B}[n^{2}z-(\mathrm{nj}+l)z_{F}]-\frac{k_{B}}{k_{A}}\frac{\delta_{A1}[n\left(z-jz_{F}/n\right)]}{n}\right]-\delta_{B1}\left(z\right),\\ \left(0\leq z\leq z_{F}\right) \end{array}$$

(2) Self-calibration at Round *m* (*m* ≥ 2)

**Figure 4:**
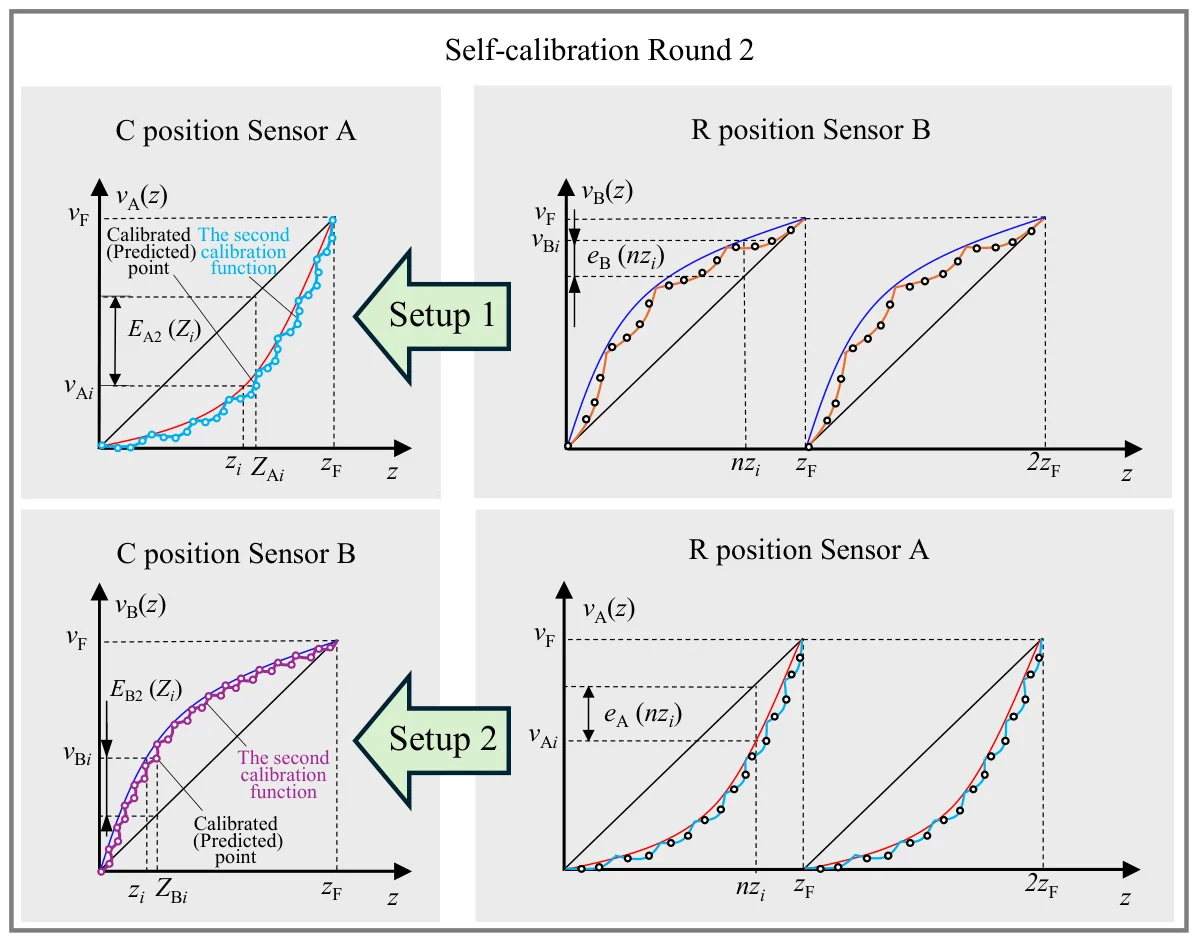
Self-calibration process of Round 2.

The same calibration operations are then repeated in the subsequent calibration rounds to get updated calibration functions. Figure 4 shows a schematic of Round 2. It can be seen that the calibration errors of Sensors A and B are reduced from those in Figure 3 of Round 1. By repeating the self-calibrations by *m* rounds, the *m*th predicted calibration functions, i.e., the final predicted calibration functions of Sensors A and B can be obtained as follows, respectively.

(20) $$v_{Am}\left(z\right)=k_{A}z+\sum_{j=0}^{\left\lceil n\right\rceil -1}E_{Am,j+1}\left(z-jz_{F}/n\right)+\delta_{Am}\left(z\right),\;\;(0\leq z<z_{F})$$

(21) $$v_{Bm}\left(z\right)=k_{B}z+\sum_{j=0}^{\left\lceil n\right\rceil -1}E_{Bm,j+1}\left(z-jz_{F}/n\right)+\delta_{Bm}\left(z\right),\;\;(0\leq z<z_{F})$$

where *δ*_A*m*_ and *δ*_B*m*_ are the error components arising in the fitting process of the discrete data for getting the *m*th predicted calibration functions of Sensors A and B, respectively. Here, *E*_A*m*,1_ and *E*_B*m*,1_ are the *m*th predicted linearity error components of Sensors A and B over their respective first calibration ranges, respectively.

The following equations describe how the calibration errors propagate between the two sensors in successive rounds over the full measurement range:

(22) $${\Delta e}_{Am}\left(z\right)=\frac{k_{A}}{nk_{B}}\sum_{j=0}^{\left\lceil n\right\rceil -1}{\Delta e}_{B,m-1}\left(\mathrm{nz}-jz_{F}\right)-\delta_{Am}\left(z\right),\;\;m\geq 2,\;\;\;0\leq z<z_{F}$$

(23) $${\Delta e}_{Bm}\left(z\right)=\frac{k_{B}}{nk_{A}}\sum_{j=0}^{\left\lceil n\right\rceil -1}{\Delta e}_{A,m}\left(\mathrm{nz}-jz_{F}\right)-\delta_{Bm}\left(z\right),\;\;m\geq 1,\;\;\;0\leq z<z_{F}$$

Each summand is restricted to its corresponding complete or partial calibration range and is taken as zero outside that range. Starting from Δ*e*_A1_(*z*) in Equation (15), these relations apply successively to all calibration rounds, including those with a non-integer magnification ratio.

Denote the final calibration error of Sensor A over its first subinterval of first calibration range of 0 ≤ *z* < *z*_F_/*n*^2*m*−1^, which is the difference between the real linearity error *e*_A,1_(*z*) and the predicted linearity error *E*_A*m*,1_ by Δ*e*_A*m*,1_(*z*). Similarly, denote the final calibration error of Sensor B over its first subinterval of first calibration range of 0 ≤ *z* < *z*_F_/*n*^2*m*^, which is the difference between the real linearity error *e*_B_(*z*) and the predicted linearity error *E*_B*m*,1_ by Δ*e*_B*m*,1_(*z*). The calibration errors Δ*e*_A*m*,1_(*z*) and Δ*e*_B*m*,1_(*z*) of Sensors A and B can be written as:

(24) $${\Delta e}_{Am,1}\left(z\right)=\frac{k_{A}}{k_{B}}\frac{e_{B}\left(n^{2m-1}z\right)}{n^{2m-1}}-\Delta_{Am,1}\left(z\right),0\leq z<z_{F}/n^{2m-1}$$

(25) $${\Delta e}_{Bm,1}\left(z\right)=\frac{e_{B}(n^{2m}z)}{n^{2m}}{-\Delta }_{Bm,1}\left(z\right),0\leq z<z_{F}/n^{2m}$$

where Δ_A*m*,1_(*z*) and Δ_B*m*,1_(*z*) are the corresponding fitting error components caused by *δ*_A*g*_ and *δ*_B*g*_, respectively, where *g* = 1, 2, …, *m*. δ_A*g*_ and δ_B*g*_ are the error components arising in the fitting process of the discrete calibration data set over the full calibration range from 0 to *z*_F_ for getting the *g*th predicted calibration functions of Sensors A and B in the *g*th calibration round, respectively. Δ_A*m*,1_(*z*) and Δ_B*m*,1_(*z*) can be expressed by

(26) $$\Delta_{Am,1}\left(z\right)=\sum_{g=1}^{m-1}\left(\frac{\delta_{Ag}\left(n^{2(m-g)}z\right)}{n^{2(m-g)}}+\frac{k_{A}}{k_{B}}\frac{\delta_{Bg}\left(n^{2(m-g)-1}z\right)}{n^{2(m-g)-1}}\right)+\delta_{Am}\left(z\right),\;0\leq z<z_{F}/n^{2m-1}$$

(27) $$\Delta_{Bm,1}\left(z\right)=\sum_{g=1}^{m}\left(\frac{k_{B}}{k_{A}}\frac{\delta_{Ag}\left(n^{2\left(m-g\right)+1}z\right)}{n^{2\left(m-g\right)+1}}+\frac{\delta_{Bg}\left(n^{2(m-g)}z\right)}{n^{2(m-g)}}\right),\;\;\;0\leq z<z_{F}/n^{2m}$$

As shown in Equations (24) and (25), the calibration error contains a contribution from the initial reference sensor error and a contribution from fitting errors introduced in successive rounds. The former rapidly decays to near zero as the number of rounds increases, whereas the latter is not necessarily eliminated by additional calibration rounds. More importantly, the calibration data sets of Sensors A and B obtained in the *m*th calibration round contain repeated patterns arising from the propagated linearity-error components. For integer *n*, the number of repetitions of the initial reference-sensor error pattern increases with *m* as *n*^2*m*−1^ for Sensor A and *n*^2*m*^ for Sensor B. For non-integer *n*, each complete calibration range repeats the preceding full-range error pattern, including any incomplete cycle inherited from the preceding step, while the final partial calibration range contains only an incomplete repetition of that pattern. This brings a great challenge to fit the discrete calibration data sets for getting the predicted calibration functions in each calibration round. From this point of view, the fitting error component is regarded as one of the most potential error sources for the self-calibration method. It should be noted that this is an important finding of this paper, which had not been addressed by Kiyono [36]. Using Artificial Neural Network fitting to reduce the fitting error components is therefore implemented in this paper as an improvement over the conventional least-squares polynomial fitting employed in Kiyono’s work [36].

In addition to the fitting error components, the sensitivity coefficients *k*_A_ and *k*_B_, the magnification ratio *n*, and the sampling number *N* are possible factors that could influence the evaluation accuracy of the linearity errors. The thermal drifts of the sensor outputs could also be an influencing factor. The influences of these factors, which have not been discussed in Kiyono’s work [36], will be investigated by computer simulation in the next section.

## 3. Investigation of Influencing Factors by Simulation

This section starts with a simulation in Section 3.1 under ideal conditions without considering any errors caused by the influencing factors shown at the end of Section 2. Then, simulations are made to investigate the influences of magnification ratio and sampling number, sensitivity mismatch, sensor noise and thermal drift in Section 3.2, Section 3.3 and Section 3.4. Finally, the effect of using an Artificial Neural Network (ANN) to reduce the residual errors caused by least-squares polynomial fitting in the self-calibration results is investigated in Section 3.5.

### 3.1. Simulation Model and Convergence Under Ideal Conditions

Firstly, mathematical models of the displacement sensors used in the simulation were established. Based on the specifications of actual displacement sensors, Sensors A and B were both assigned a measurement range of 100 μm and a corresponding output voltage range of 20 V. The ideal output of each sensor was assumed to be a straight line on which an error component was added as the linearity error component as shown in Equations (2) or (3). The added linearity error functions for Sensors A and B were defined as follows, where *z* is expressed in μm and the resulting error is expressed in mV:

(28) $$e_{A}(z)=38[\cos(\frac{2\pi z}{100})-1]+4[\cos(\frac{6\pi z}{100})-1]-5\sin(\frac{12\pi z}{100})+3\sin(\frac{14\pi z}{100})$$

(29) $$e_{B}(z)=-\{88[\cos(\frac{2\pi z}{100})-1]+5[\cos(\frac{6\pi z}{100})-1]-8\sin(\frac{12\pi z}{100})-5\sin(\frac{14\pi z}{100})\}$$

**Figure 5:**
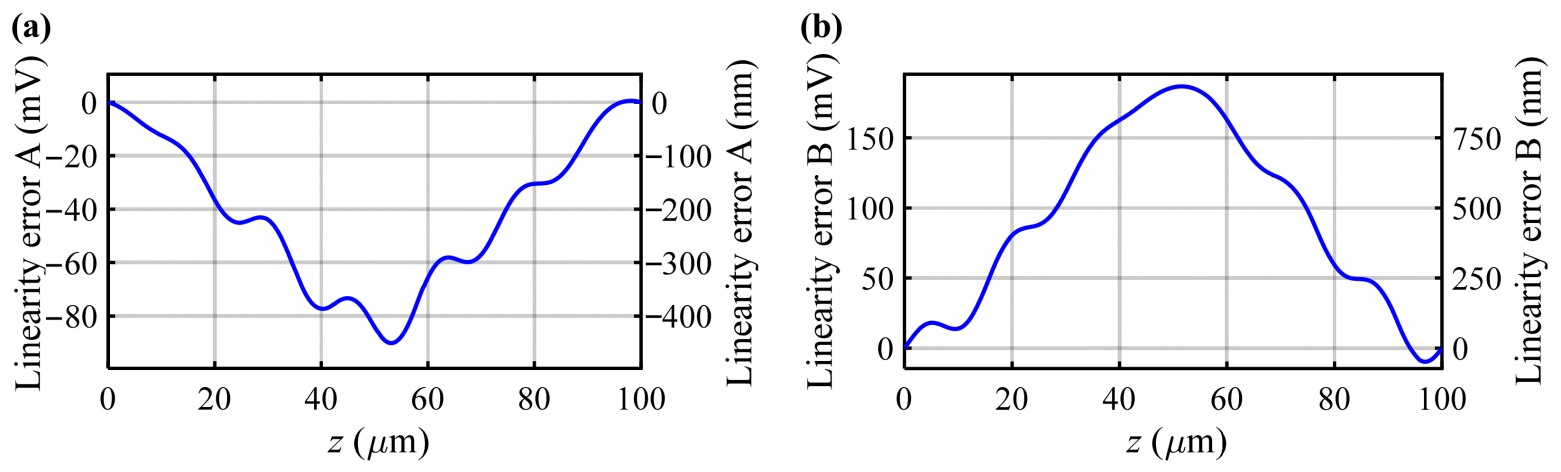
Designed linearity error curves of Sensors A and B. (**a**) Sensor A. (**b**) Sensor B.

The integer harmonic components in Equations (28) and (29) make both linearity error curves equal to zero at the two ends of the measurement range. The linearity errors of Sensors A and B are shown in Figure 5a and Figure 5b, respectively. As shown in the figures, Sensor A has a minimum of −90.16 mV, whereas Sensor B has a maximum of 186.63 mV near the center of the measurement range.

The self-calibration procedure shown in Section 2 was then applied under ideal conditions. The magnification ratios were identical at the two setups and were set to *n*_A_ = *n*_B_ = 3.5. Similarly, the identical sensitivity coefficients were set as *k*_A_ = *k*_B_ = 0.2 V/μm. The number of sampling points was set as *N* = 2000. The highest degree of least-squares polynomial fitting was set to be 20, which was high enough to fit the linearity errors shown in Figure 5, while avoiding the numerical ill-conditioning associated with higher-degree least-squares polynomial fitting [50].

**Figure 6:**
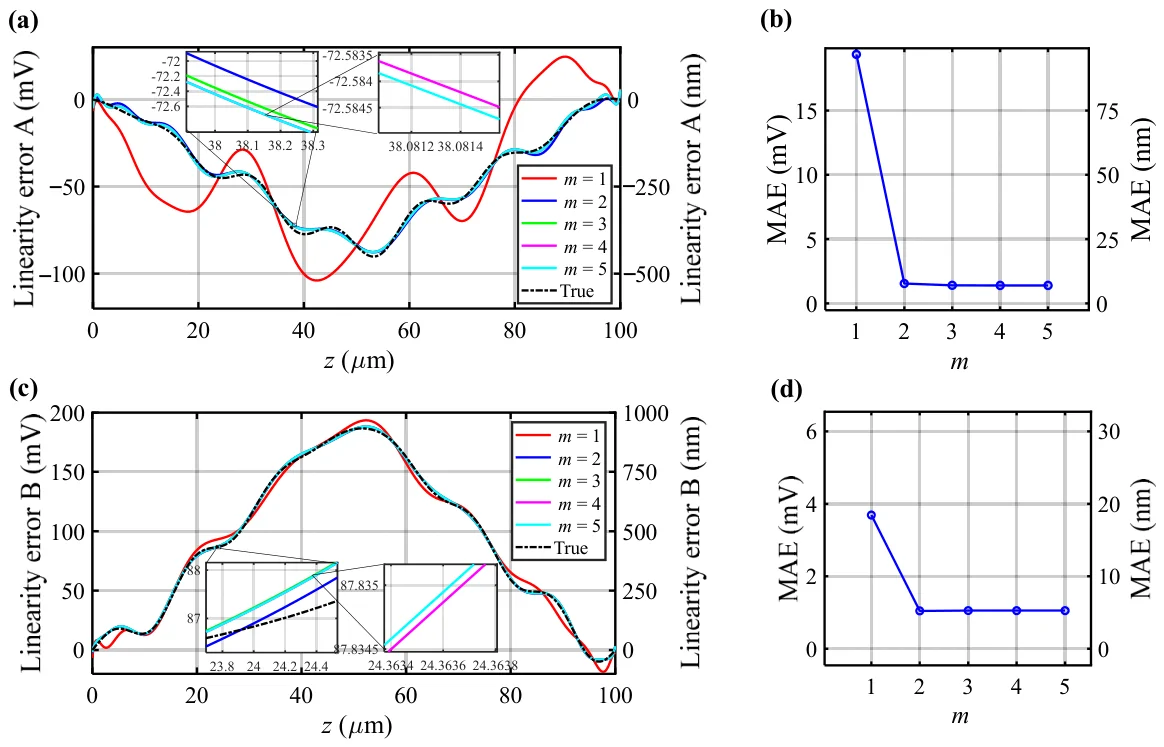
Self-calibration simulation under ideal conditions. (**a**) Estimated linearity error curves of Sensor A over five rounds. (**b**) MAE of Sensor A. (**c**) Estimated linearity error curves of Sensor B over five rounds. (**d**) MAE of Sensor B.

Figure 6a,c show the linearity error curves of Sensors A and B, respectively, estimated after each of the five calibration rounds. Figure 6b,d show the corresponding mean absolute error (MAE) between the estimated and true linearity error curves over the five rounds. As the number of self-calibration rounds increases, the estimated curves rapidly approach the true curves. The MAE of Sensor A becomes nearly constant after Round 4, whereas that of Sensor B stabilizes earlier. Therefore, the maximum number of self-calibration rounds, *m*, was set to 5 in all subsequent analyses and experiments to ensure convergence.

(30) $$\mathrm{MAE}=\frac{1}{z_{F}}\int_{0}^{z_{F}}|E(z)-e(z)|\mathrm{dz}$$

The MAE used in this comparison is defined by Equation (30), where *E*(*z*) is the linearity error component obtained after the *m*th calibration round and *e*(*z*) is the defined true error. For numerical evaluation, the integral was discretized using 20,000 uniformly spaced points over the full measurement range. The same discretization was used for all subsequent MAE calculations.

### 3.2. Influences of Magnification Ratio and Sampling Number

The derivation in Section 2 applies to both integer and non-integer magnification ratios. In the numerical implementation, *n* is estimated from the measured voltage data, accounting for the complete calibration ranges and any final partial range. Let

*H* = ⌊*n*⌋,*η* = *n* − *H*,*J* = ⌈*n*⌉ (31)

where *H* is the number of complete voltage segments, *η* is the fractional part of *n*, and *J* is the total number of voltage segments. The block index is therefore *j* = 0,1,…,*J* − 1.

For a non-integer *n*, the first *H* blocks correspond to complete output sweeps of the sensor at Position R from 0 to *v_F_*, whereas the final segment contains only the remaining voltage data:

(32) $$0\leq v_{R,i}^{\left(j\right)}\leq \left\{\begin{array}{cc} v_{F}, & j=0,1,\ldots ,H-1,\\ v_{R,\mathrm{end}}, & j=H. \end{array}\right.$$

Here, *v*_R,end_ is the final output of the sensor at Position R in the last partial block. Therefore, the first *H* complete blocks are fixed, and only the final block is truncated.

Because each complete sweep contributes one full output range, the magnification ratio is calculated as

(33) $$\hat{n}=H+\frac{v_{R,\mathrm{end}}}{v_{F}}$$

According to the sensor model

*v*_R_(*z*) = *k*_R_*z* + *e*_R_(*z*) (34)

the output change in the final block is

*v*_R,end_ = *ηv*_F_+Δ*e*_R,end_ (35)

where

Δ*e*_R,end_ = *e*_R_(*ηz*_F_) − *e*_R_(0) (36)

is the change in the linearity error component between the beginning and end of the final block. Therefore, the relative error in the calculated magnification ratio is

(37) $$\frac{\hat{n}-n}{n}=\frac{\Delta e_{R,\mathrm{end}}}{nv_{F}}$$

For *n* = 3.5, four voltage blocks are used: three complete blocks and one partial block ending at approximately 0.5*v*_F_. If the linearity error at this endpoint is 1% of *v*_F_, the relative error in the complete magnification ratio is 0.29%. The propagated calibration points are also shifted relative to the original calibration points by a factor of

(38) $$\frac{{\hat{Z}}_{i}-Z_{i}}{Z_{i}}=\frac{n}{\hat{n}}-1=\frac{n-\hat{n}}{\hat{n}}$$

Although this deviation theoretically affects the calibration result, its effect is limited and systematic for a given magnification ratio.

Additionally, the effect of the number of sampling points *N* was not quantitatively examined in Section 2. In addition, the actual magnification ratio *n*_true_ may deviate from its designed value because of manufacturing and installation errors in the fixture. To investigate the influence of these two parameters, a two-parameter sweep was conducted over the common magnification ratio *n*_true_, ranging from 2.5 to 4.5, while *N* was varied from 50 to 2000.

**Figure 7:**
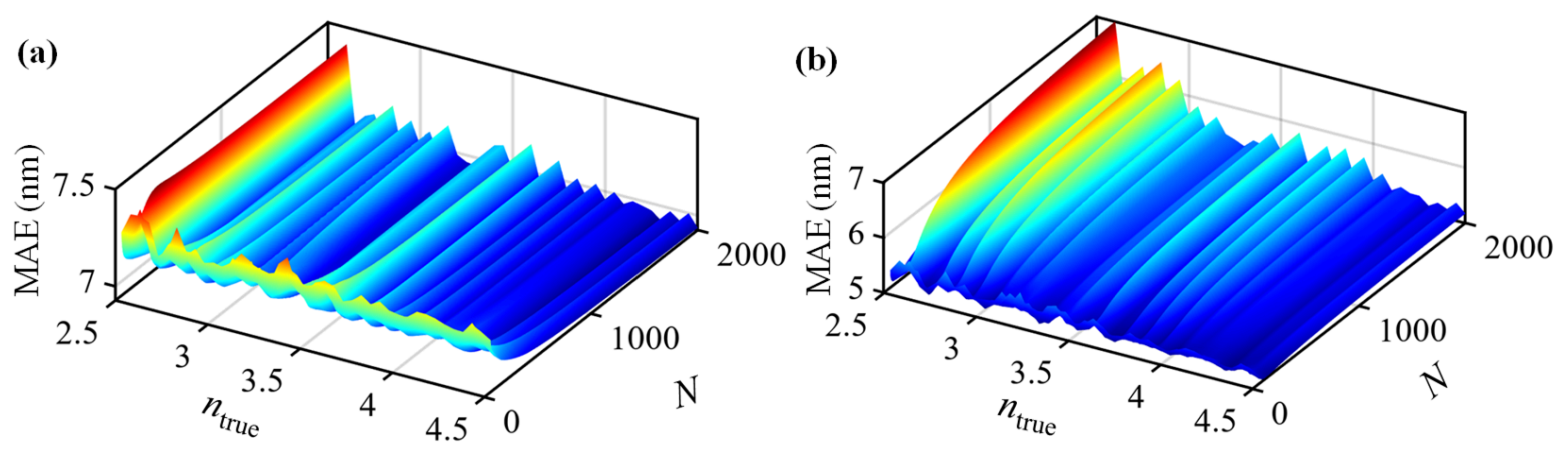
Influence of the sampling number N and the common actual magnification ratio on the fifth-round MAE. (**a**) Sensor A. (**b**) Sensor B.

The calibration MAE was calculated for each combination of *n*_true_ and *N*. The results are shown in Figure 7.

When *N* is small, the discrete calibration data are relatively sparse, causing the polynomial fitting results and the corresponding MAEs to vary with *N*. Once *N* reaches approximately 500, further increases have little effect on the MAE. Over the tested range of *n*_true_, the variation in MAE remains below 1 nm. Therefore, for the error curves and fitting settings used in this study, *N* = 500 provides a practical balance between data volume and stable fitting accuracy. Beyond this sampling density, neither additional sampling points nor small changes in the common magnification ratio are the main factors limiting calibration accuracy.

**Figure 8:**
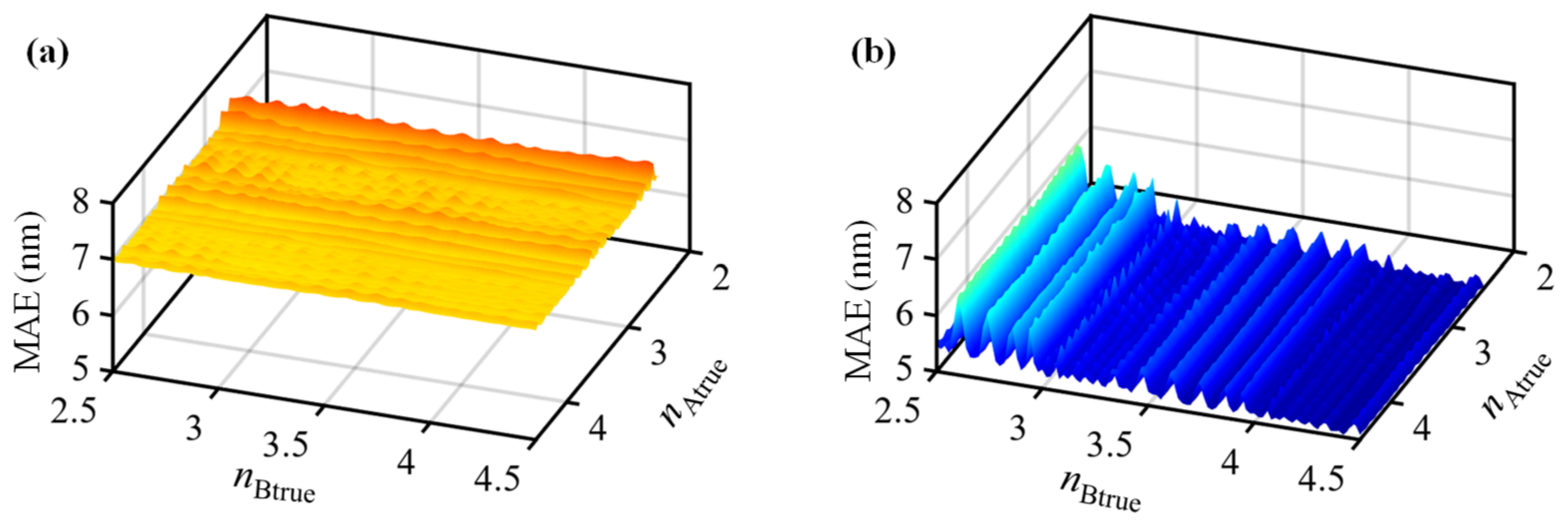
Influence of unequal actual magnification ratios (**a**) Fifth-round MAE of Sensor A. (**b**) Fifth-round MAE of Sensor B.

The magnification ratios in the two setups of each calibration round may be different from each other. Therefore, the simulation varied the magnification ratios used to calibrate Sensor A (*n*_Atrue_) and Sensor B (*n*_Btrue_), independently from 2.5 to 4.5. The simulation results in Figure 8 show that the MAE of each sensor is more sensitive to the magnification ratio used when that sensor is calibrated, resulting in the different trends of the two MAE results. Nevertheless, the variation in MAE remains below 1 nm. Thus, a mismatch between the two setups does not prevent convergence.

### 3.3. Influence of Sensitivity Mismatch

The derivation in Section 2 assumes that the sensitivity coefficients *k*_A_ and *k*_B_ are known. In practice, however, the actual sensitivity may differ slightly from the nominal value used by the calibration algorithm. To evaluate this effect, the assumed sensitivities were fixed at 0.200 V/μm, while each actual sensitivity varied from 0.198 to 0.202 V/μm, corresponding to ±1%.

**Figure 9:**
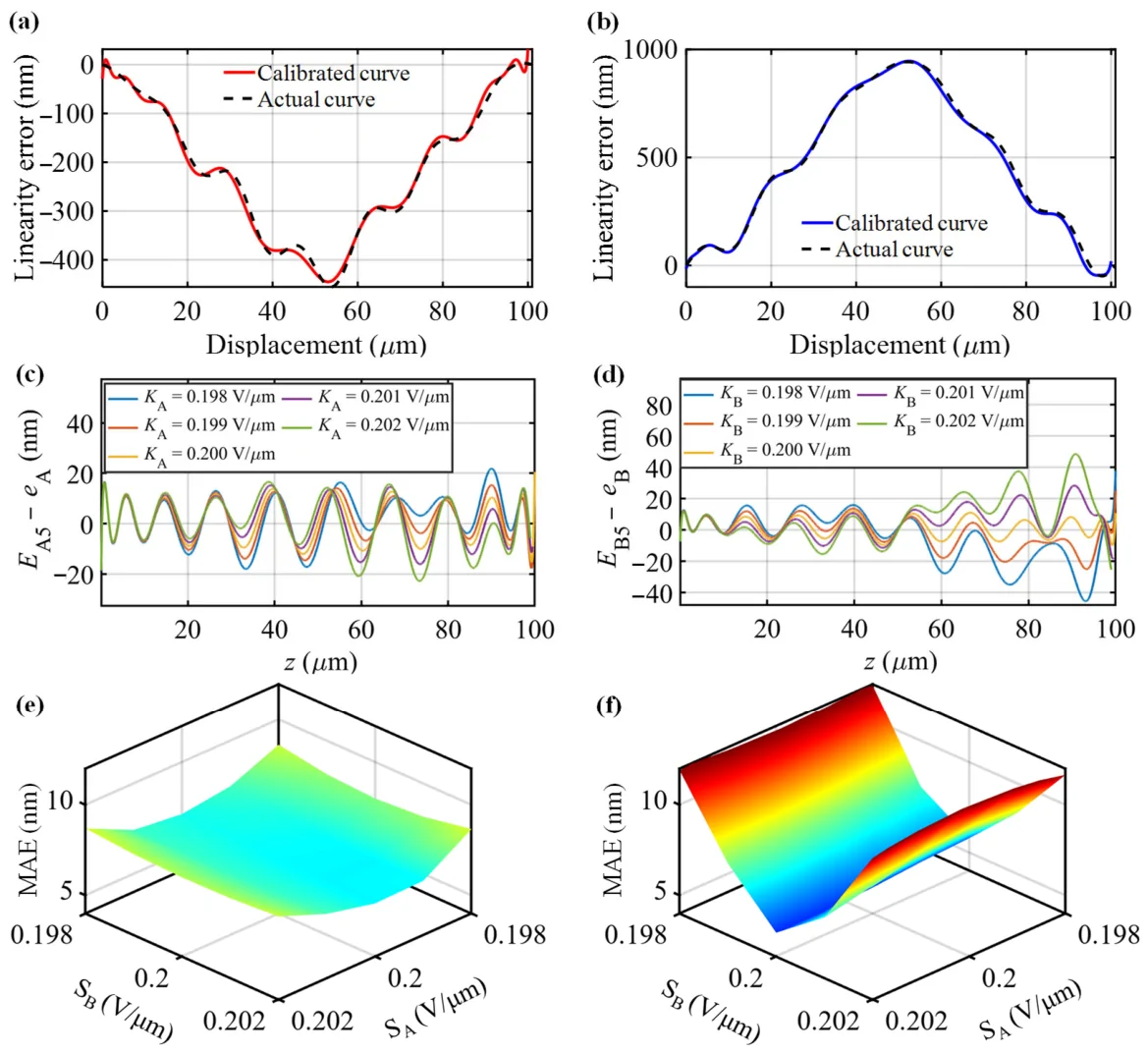
Influence of sensitivity mismatch. (**a**) Linearity error result of Sensor A. (**b**) Linearity error result of Sensor B. (**c**) Fitting residual result of Sensor A. (**d**) Fitting residual result of Sensor B. (**e**) Fitting residual MAE result of Sensor A. (**f**) Fitting residual MAE result of Sensor B.

Figure 9a,b compare the predicted and true linearity error curves when one of *k*_A_ and *k*_B_ deviates from the nominal value, respectively, while the other remains at 0.200 V/μm. Figure 9c,d show the pointwise differences between the estimated and true linearity error curves at several actual sensitivities. It can be observed that the deviation increases as the measurement position approaches the maximum value.

Figure 9e,f show the MAE sweeping results when the actual sensitivity coefficients of the two sensors vary independently. The minimum MAE occurs near the nominal sensitivity pair and increases smoothly toward the boundaries of the tested range. The MAE of each sensor is more sensitive to a deviation in its own sensitivity coefficient. Sensor B is affected more strongly because its true linearity error has a larger maximum value. Nevertheless, the increase in MAE remains limited to several nanometers over the tested range. Thus, moderate sensitivity errors reduce the calibration accuracy but do not prevent convergence of the self-calibration process.

### 3.4. Influence of Sensor Noise and Thermal Drift

**Figure 10:**
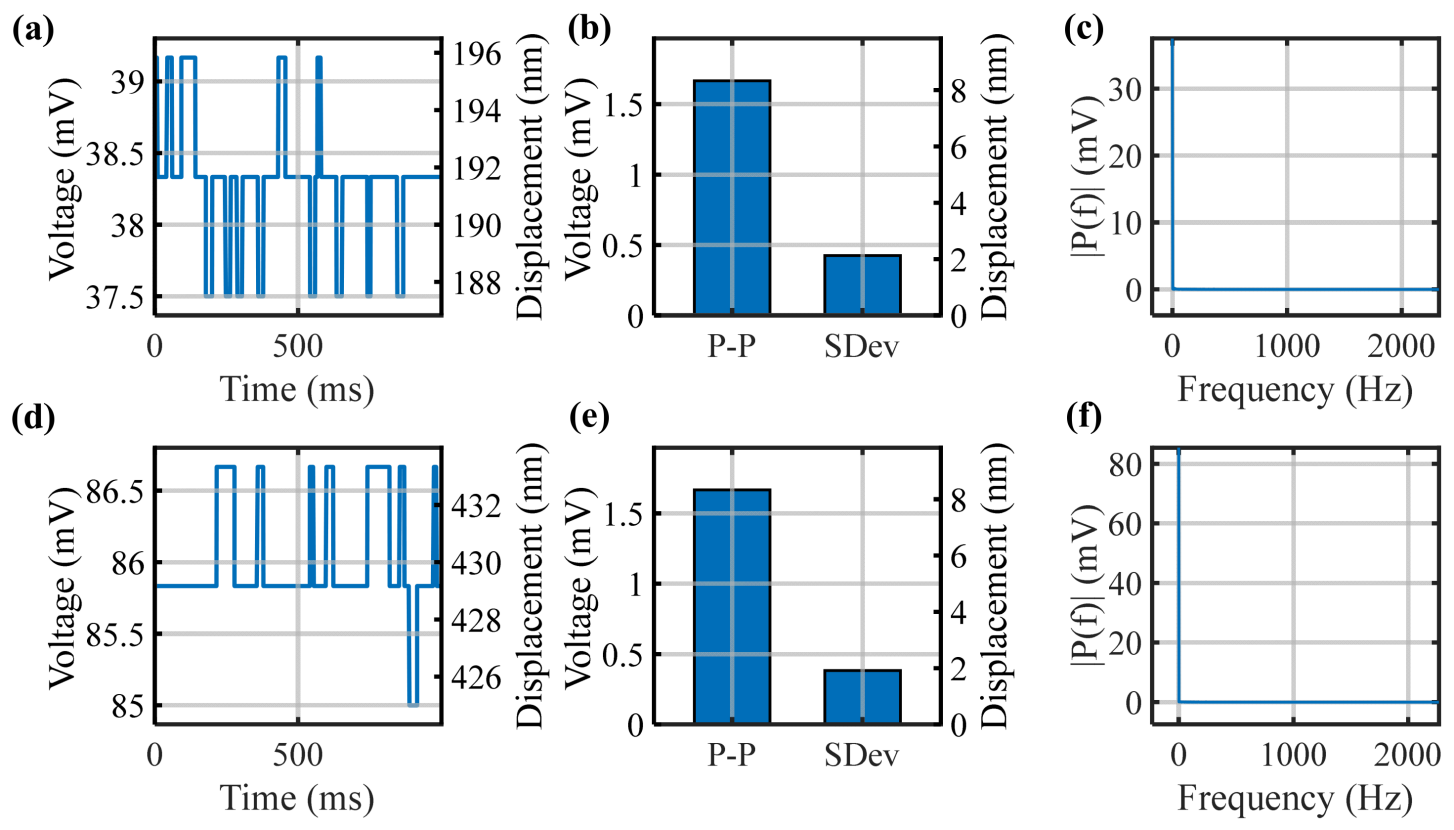
Short-term noise characteristics of the two sensors at 100 kS/s. (**a**) 1 s raw noise data of Sensor A. (**d**) 1 s raw noise data of Sensor B. (**b**) peak-to-peak value and standard deviation of Sensor A’s noise data. (**e**) peak-to-peak value and standard deviation of Sensor B’s noise data. (**c**) FFT result of Sensor A’s noise data. (**f**) FFT result of Sensor B’s noise data.

Before modeling drift, the short-term output stability of the two actual capacitive displacement sensors was measured for 1 s at a sampling rate of 100 kS/s. Figure 10 shows the raw signals, peak-to-peak values, standard deviations, and frequency spectra. Both sensors exhibit a peak-to-peak displacement fluctuation of approximately 8 nm and a standard deviation of approximately 2 nm. The spectra contain little high-frequency content. Because 500 points are used for each self-calibration data set, this short-term random noise can be reduced by averaging and is smaller than the long-term drift considered below.

**Figure 11:**
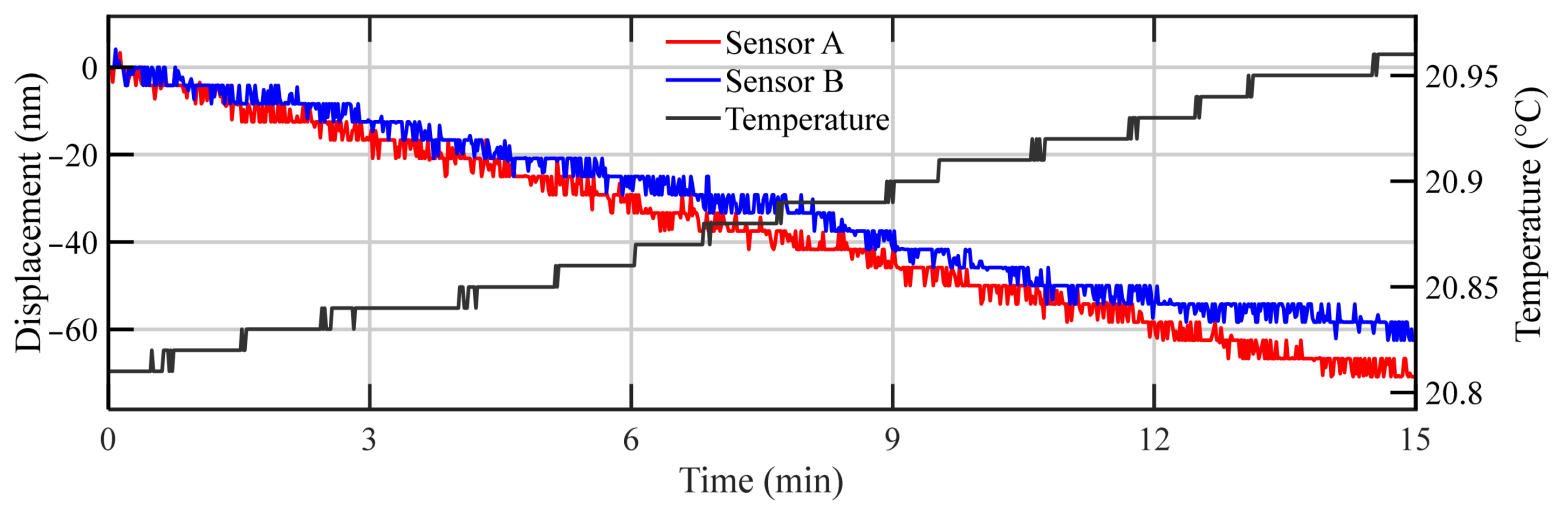
15 min drift of temperature and sensor outputs.

Figure 11 shows the outputs of both sensors and the measured temperature over 15 min. As the temperature gradually increases, both sensor outputs drift in the same direction. This common trend suggests that the sensor heads, electronics, fixture, and surrounding structure are affected by the changing thermal conditions. Unlike random noise, this slow drift is correlated with the sampling order of the calibration points and cannot be removed by simple averaging.

The previous manual setup proposed by Kiyono [36] required approximately 1 h for one complete self-calibration sampling process. Assuming a constant drift rate, linear extrapolation of the measured 15 min trend yields same-direction drift of 240 nm over 1 h. To simulate this effect, a time-dependent drift term was added to each calibration point according to its acquisition time. Because the two sensor setups are sampled at different times, the drift is converted into different position-dependent errors during data transformation and fitting.

**Figure 12:**
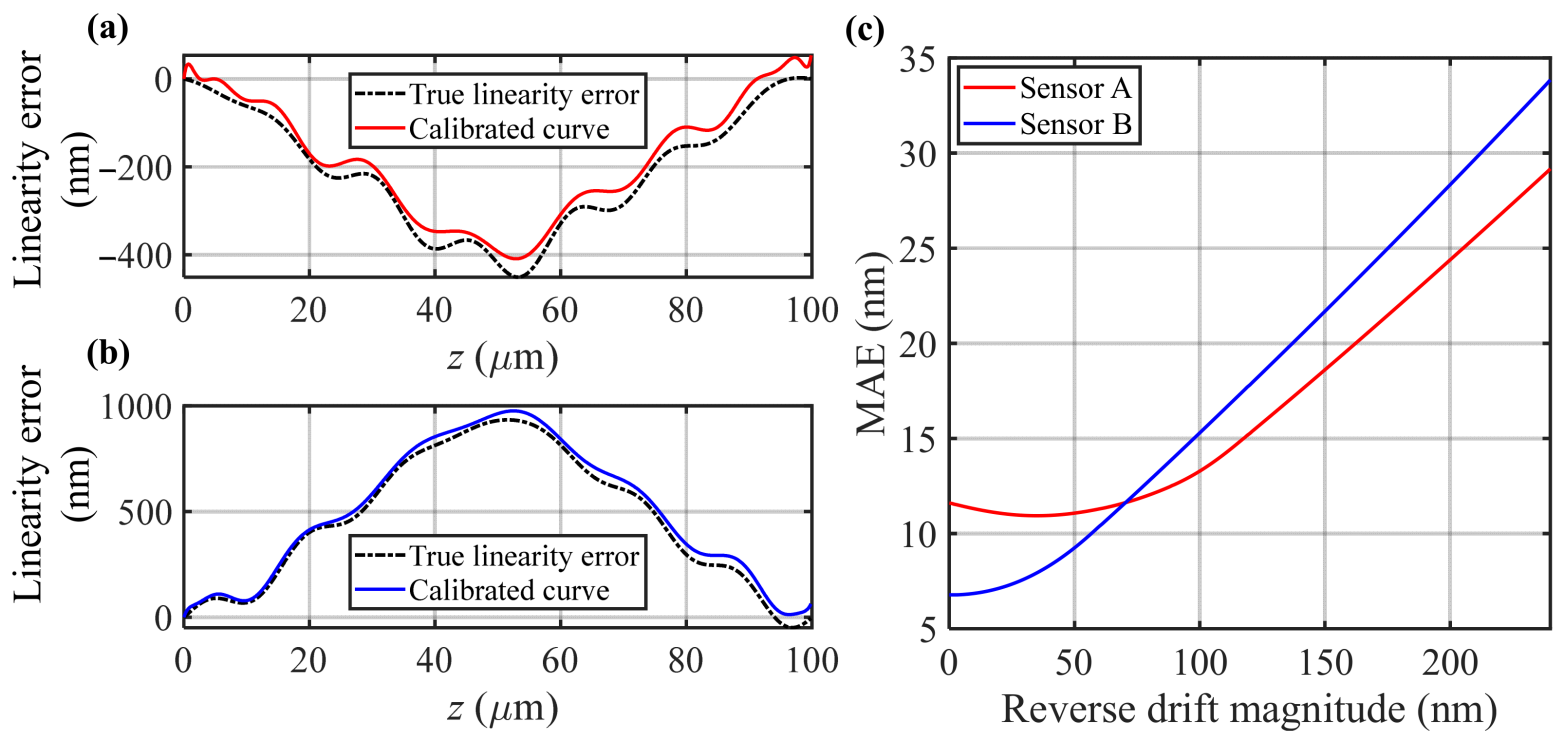
Simulation results including time-dependent drift. (**a**) Sensor A with 240 nm drift. (**b**) Sensor B with 240 nm drift. (**c**) MAE between calibrated curve and true curve as a function of drift magnitude.

Figure 12a,b show that a drift of 240 nm produces deviations between the predicted and true linearity error curves that are much larger than those under ideal conditions. Figure 12c shows that the MAE increases with the drift magnitude, reaching approximately 29 nm for Sensor A and 34 nm for Sensor B for a drift of 240 nm. Therefore, reducing the calibration time and stabilizing the thermal environment are essential for high-accuracy self-calibration.

### 3.5. Reduction of Polynomial Fitting Errors by Artificial Neural Network

In this subsection, the fitting results by the Artificial Neural Network fitting method are compared with those by the conventional least-squares polynomial fitting method, which had been employed in the previous work by Kiyono [36]. The comparison examines their ability to fit the high-frequency components generated in the calibration data through repeated self-calibration. Since identifying the best fitting method is beyond the scope of this paper, the comparison is limited to these two methods under the simulated conditions.

The analysis in Section 2 indicates that the first terms in Equations (24) and (25) decrease as the number of calibration rounds *m* increases, whereas the fitting-error terms generated in each round remain in the final result. To verify this mathematical derivation, a simulation was performed with *N* = 500, *n*_A_ = *n*_B_ = 3.5, *k*_A_ = *k*_B_ = 0.2 V/μm, *m* = 5 without noise or drift.

**Figure 13:**
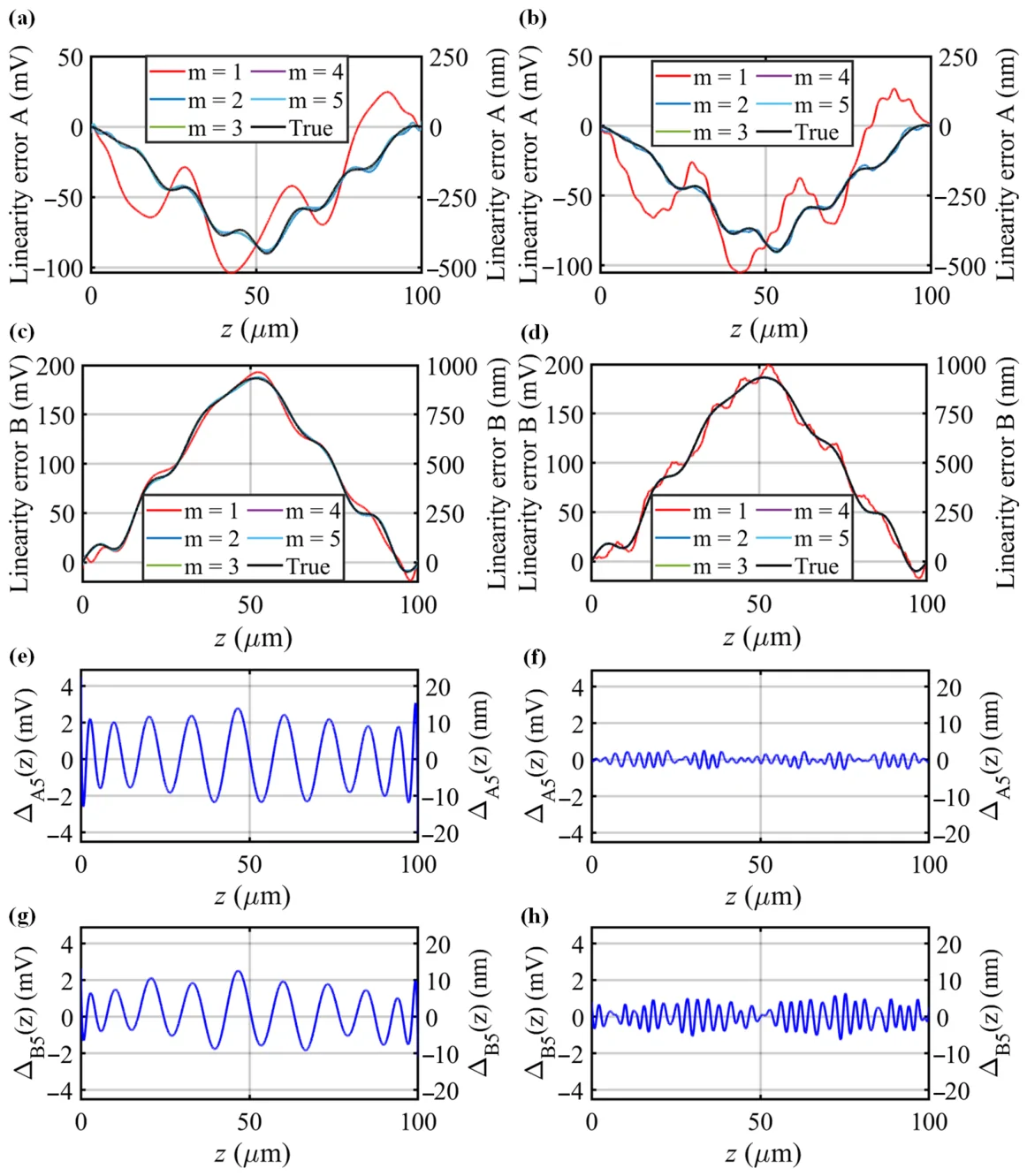
Comparison of fitting methods. (**a**) Calibration result of Sensor A by polynomial. (**b**) Calibration result of Sensor A by ANN. (**c**) Calibration result of Sensor B by polynomial. (**d**) Calibration result of Sensor B by ANN. (**e**) Δ_A5_(*z*) result of Sensor A by polynomial. (**f**) Δ_A5_(*z*) result of Sensor A by ANN. (**g**) Δ_B5_(*z*) result of Sensor B by polynomial. (**h**) Δ_B5_(*z*) result of Sensor B by ANN.

Figure 13a,c show the five-round results obtained using 20th-order conventional least-squares polynomial fitting. Although the main low-frequency shapes of the true linearity error curves are recovered, the fifth-round curves retain broad and smooth residuals over much of the measurement range. Figure 13e,g show the corresponding accumulated fitting-error terms Δ_A5_ and Δ_B5_. These terms are calculated by subtracting the fifth-round polynomial fitting result from the corresponding reference curves generated by an independent 20,000-point linear interpolation. The 20,000-point curve was regarded as the true calibrated curve. The final fitting curve waveforms closely match the remaining residuals, indicating that the fitting errors are position-dependent and remain in the final calibration result.

An Artificial Neural Network (ANN) was then introduced in the fitting process of each self-calibration round to reduce Δ_A*m*_ and Δ_B*m*_. As predicted by Equations (26) and (27), repeated range segmentation increases the spatial-frequency content of the discrete calibration data. A fixed global least-squares polynomial must balance the overall trend and these localized variations, whereas a multilayer nonlinear model provides greater local fitting flexibility. For direct comparison, an ANN was trained independently for each sensor and each calibration round using the same (*N* = 500) discrete sampling data. The ANN consisted of one input neuron, three hidden layers containing 32, 16, and 8 neurons, and one output neuron. The hyperbolic tangent function was used in the hidden layers, and the network was trained using the scaled conjugate-gradient algorithm optimizer with the mean squared error (MSE) as the loss function. For each sensor and calibration round, the 500 sampling points were sorted by displacement and divided into 50 groups of 10 consecutive points. One point from each group was selected for validation using a fixed random seed, giving 50 validation points and 450 training points. Both endpoints were retained for training. The validation points were excluded from weight updates. Their predicted linearity errors were compared with the values derived from the calibration data. Validation MSE was monitored for early stopping, and validation MAE was calculated after training. For the final-round fitting, 10-fold validation was also performed using the same grouping. Each group contributed one point to each fold, and the primary validation set corresponded to one predefined fold. Each fold was used in turn for validation, with the network retrained on the other nine folds. The overall MAE was calculated across all validation points. Spatial correlation between neighboring sampling points was not considered in this study.

The ANN fitting results are shown in Figure 13b,d, with the corresponding Δ_A5_ and Δ_B5_ shown in Figure 13f,h. Compared with least-squares polynomial fitting, the ANN follows the localized components more closely and substantially reduces both the accumulated fitting-error terms and the MAEs. The remaining errors have smaller amplitudes, vary more frequently around zero, and contain less low-frequency bias. These results show that the ANN reduces the systematic fitting error of the global least-squares polynomial while better retaining the local high-frequency features.

To quantitatively compare the two fitting methods, each fitted linearity-error curve was evaluated against the known true curve on the same displacement grid. At each evaluation position, the signed difference was defined as the true value minus the fitted value. Four metrics were calculated: mean deviation (MD), maximum absolute deviation (MAD), mean absolute error (MAE), and root mean square error (RMSE).

MD is the mean value of the signed differences and describes the average offset between the curves. Positive and negative differences can cancel, so a small MD does not necessarily indicate small overall errors. MAD is the largest absolute difference across the evaluation positions and identifies the greatest local discrepancy. MAE is the mean value of absolute differences and measures the average error magnitude without cancellation. RMSE is the square root of the mean squared differences and is more sensitive to larger errors.

**Figure 14:**
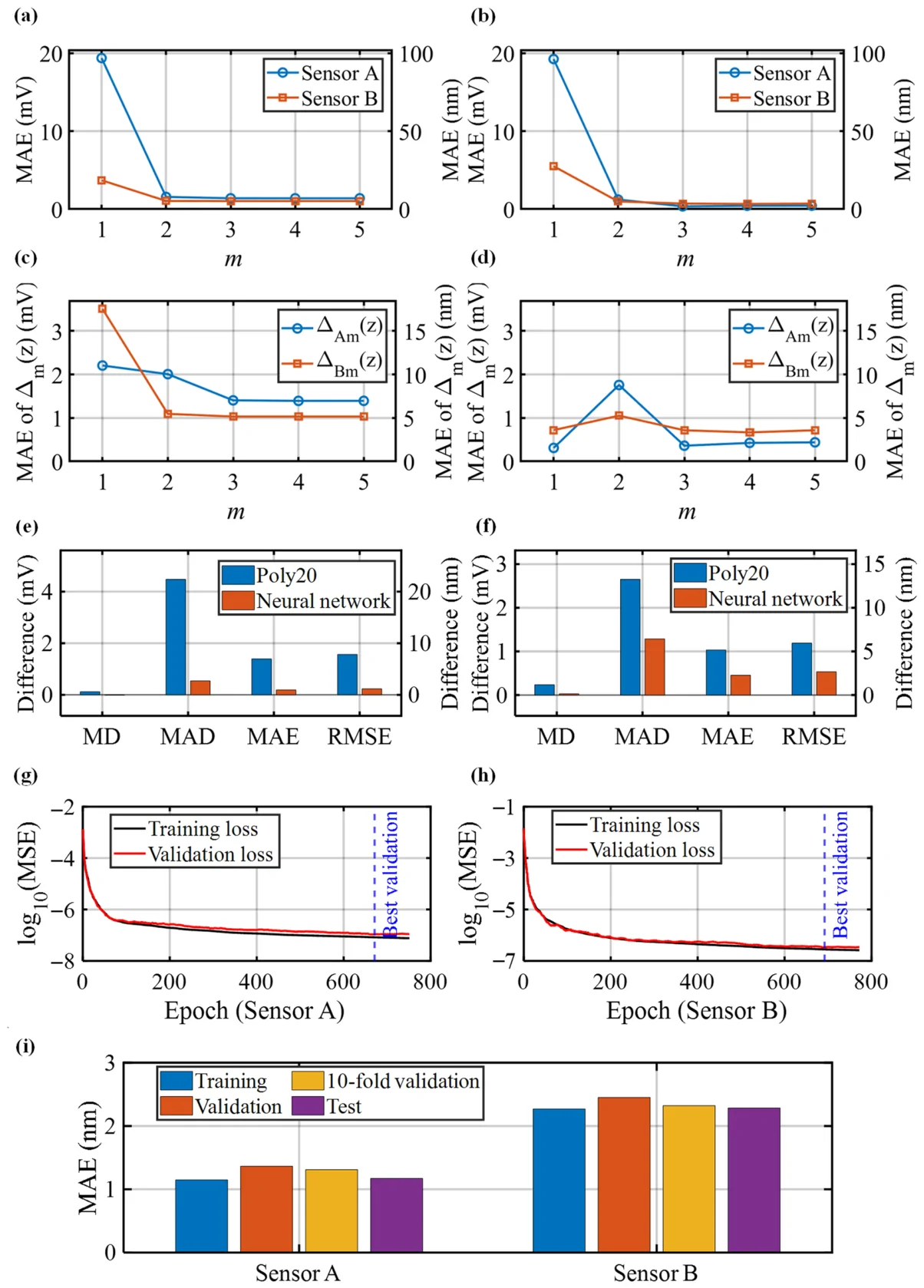
Comparison of polynomial and neural-network fitting results. (**a**) MAE result versus calibration round number m by polynomial. (**b**) MAE result versus calibration round number m by ANN. (**c**) Δ*_m_*(*z*) result versus calibration round number *m* by polynomial. (**d**) Δ*_m_*(*z*) result versus calibration round number *m* by ANN. (**e**) Final-round fitting metrics for Sensor A. (**f**) Final-round fitting metrics for Sensor B. (**g**) ANN loss for Sensor A. (**h**) ANN loss for Sensor B. (**i**) ANN validation and test performance.

Figure 14 shows that both fitting methods’ final-round calibration MAEs were almost equal to the corresponding fitting-error components Δ_A5_(*z*) and Δ_B5_(*z*). This agreement is consistent with the error-propagation analysis in Section 2. in Section 2: as shown in Equations (24) and (25), increasing the calibration round *m* causes the first residual term, which is related to the real linearity error of the sensor located at the reference position in the first calibration round, to converge toward zero; however, the second residual term, which consists of the fitting-error components arising in each calibration round, does not converge to zero and therefore becomes the main source of the final calibration error after convergence. Additionally, the consistency of the final MAEs across the training, validation, and test sets indicate that no significant overfitting occurred.

The final-round total MAEs of the 20th-order least-squares polynomial are 1.392 mV for Sensor A and 1.031 mV for Sensor B, corresponding to 6.960 nm and 5.155 nm at a sensitivity of 0.2 V/μm. The corresponding Δ_A5_(*z*) and Δ_B5_(*z*) values are the same as the total MAEs; the difference only appears at the fourth decimal place. The ANN fitting result values decreased to 0.189 mV and 0.453 mV for Sensor A and Sensor B, or approximately 0.943 nm and 2.266 nm, respectively. For each sensor, the MAE of the accumulated fitting-error component closely agrees with the corresponding final calibration MAE to three decimal places, with differences appearing only in the fourth decimal place. These results show the application of ANN effectively reduced the fitting errors that remain after convergence.

It should be noted that, in this paper, the comparison of ANN fitting was only made with the conventional least-squares polynomial fitting method, which had been employed in the previous work by Kiyono [36] since the investigation of the best fitting method is not the focus of this paper. More comparison between ANN and other advanced fitting methods such as smoothing splines, regularized polynomial fitting will be made in future work.

### 3.6. Summary of Influencing Factors and Implications for System Design

Overall, the simulations identify the conditions required for reliable convergence and the error sources that limit accuracy after convergence. These findings, summarized in Table 1, provide the basis for experimental system design.

The simulations in Section 3 support the convergence and error-propagation analysis in Section 2 and quantify practical factors not fully addressed there, including number of sampling points, magnification-ratio variations and mismatches, sensitivity mismatch, drift, and fitting method. Under the present conditions, *N* = 500 provides stable fitting accuracy, while small variations or mismatches in the magnification ratio have a limited effect. To ensure calibration accuracy, the sensitivity coefficients of both sensors should also be accurately measured. In contrast, drift and residual fitting errors are the main factors limiting calibration accuracy. The experimental system should therefore minimize the total calibration time to reduce the influence of drift. The ANN results further show that a more flexible fitting model can substantially improve the final calibration accuracy.

## 4. Experiment

Based on the findings in Section 3, an automated self-calibration setup was designed and built to reduce the calibration time. The complete self-calibration experiment was performed 5 times to evaluate its repeatability. The calibration results were then verified using a laser interferometer. Finally, an ANN was used instead of least-squares polynomial fitting to fit the same calibration data, and the results were compared with those obtained using the laser interferometer.

### 4.1. Design of the Self-Calibration System

**Figure 15:**
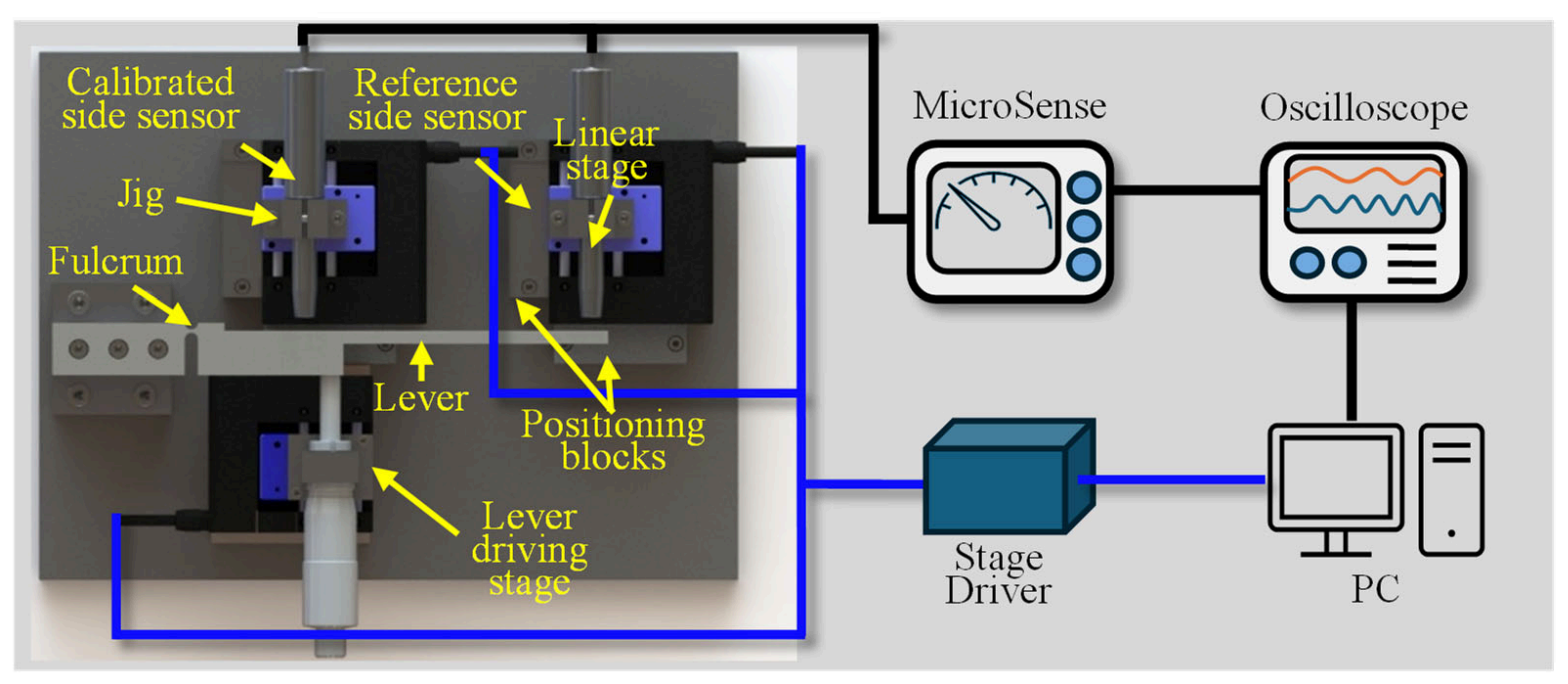
Experimental setup.

Figure 15 shows the constructed automated self-calibration setup. A linear stage (M-110.1DG1, PI, Kawasaki, Japan) was used to drive the lever’s rotation. Two capacitive displacement sensors (ADE 2102/2001K probe, JAPAN ADE Ltd., Tokyo, Japan) were positioned near and far from the rotation center (fulcrum), serving as the sensor to be calibrated and the reference sensor, respectively. All stages were controlled by a PC. The signals from the two capacitive displacement sensors were processed by a Micro Sense (MicroSense 3401 HR, JAPAN ADE Ltd., Tokyo, Japan) signal processor and then fed to an oscilloscope (DL850E, YOKOGAWA, Tokyo, Japan) for sampling and recording on the PC.

Through the design of this automated self-calibration setup, not only can the influence of manual operation be eliminated, but the calibration sampling time can also be reduced from approximately 1 h to fifteen minutes, thereby effectively shrinking the impact of drift.

### 4.2. Repeatability of the Self-Calibration Experiment

**Figure 16:**
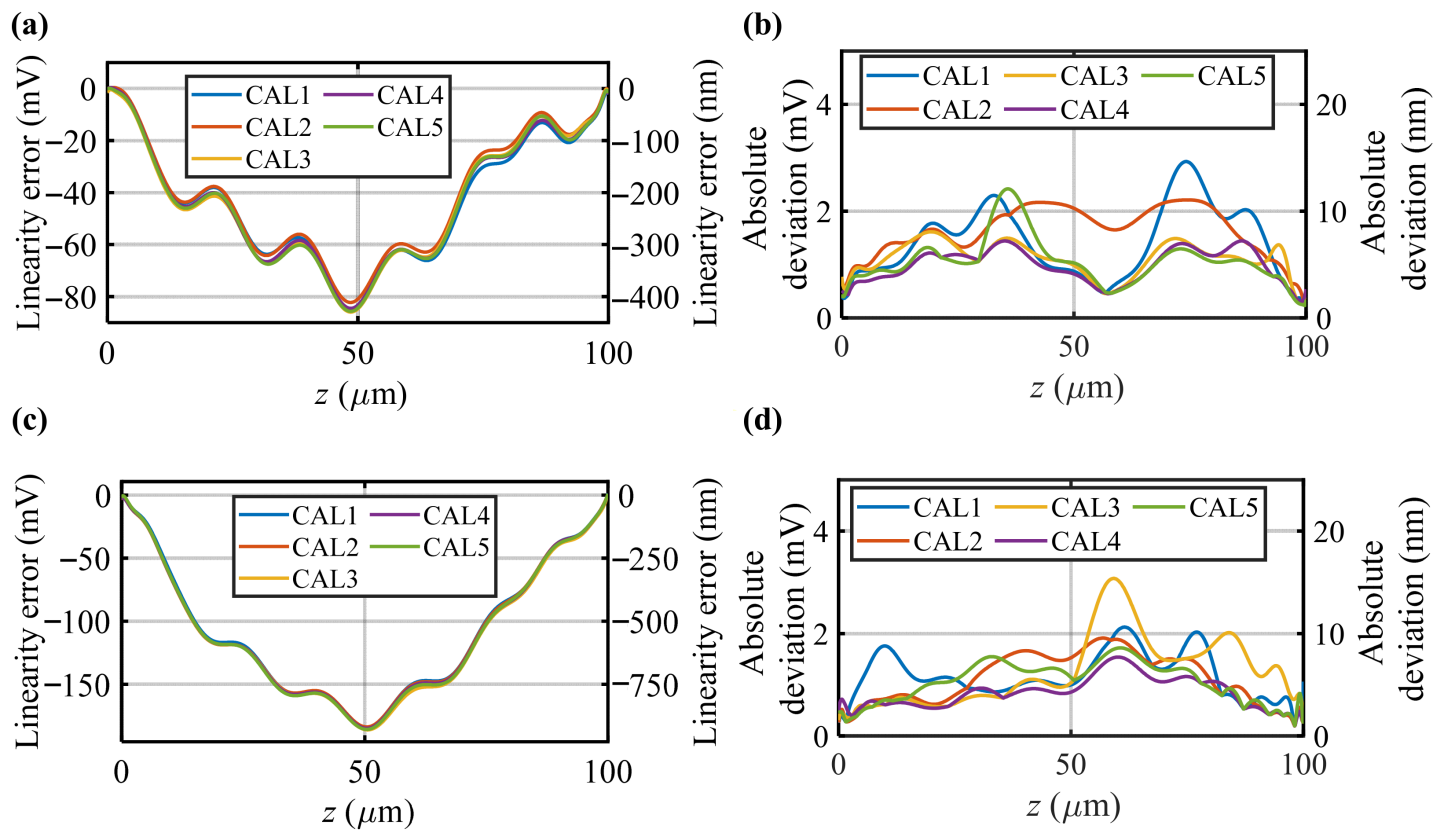
Five repeated self-calibration results using 20th-order polynomial fitting. (**a**) Linearity error curves of Sensor A. (**b**) Pointwise deviations of Sensor A. (**c**) Linearity error curves of Sensor B. (**d**) Pointwise deviations of Sensor B.

The self-calibration experiment was conducted under the same conditions and repeated 5 times. Each experiment consisted of five self-calibration rounds with 500 sampling points per data set for each sensor, and every step’s discrete calibration data was fitted using a 20th-order least-squares polynomial. The sensitivities were measured using an interferometer prior and were *k*_A_ = 0.19424 V/μm and *k*_B_ = 0.18490 V/μm, respectively. For comparison on a common displacement axis, the displacement coordinates of the calibrated linearity error curves *e*_A_(*z*) and *e*_B_(*z*) were multiplied by *k*_A_/0.2 and *k*_B_/0.2, respectively, to normalize both measurement ranges to 0–100 μm. The linearity error values remained unchanged. Figure 16a,c show the resulting linearity error curves of Sensors A and B. The five curves of each sensor nearly overlap over the full 100 μm range, including the local features around the center and near the two ends.

Figure 16b,d give the absolute deviation from the mean among the five self-calibration results. Most deviations are below 2 mV, and the largest local values are approximately 3 mV, corresponding to about 15 nm. The close agreement of the five curves confirms that the automated self-calibration setup and data processing method provide repeatable self-calibration results.

### 4.3. Verification Using a Renishaw Laser Interferometer

**Figure 17:**
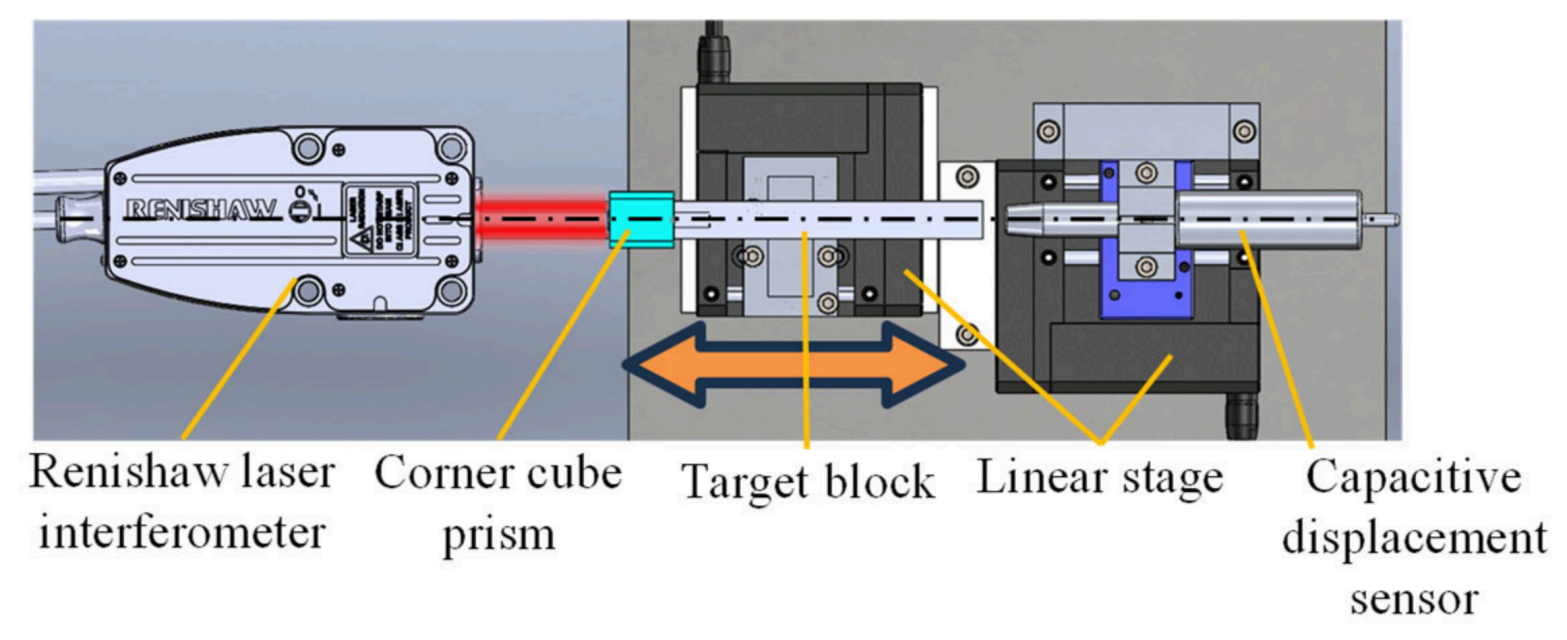
Verification setup using a Renishaw laser interferometer as the displacement reference.

To verify the reliability of the self-calibration results, the experimental setup for verification was designed and built as shown in Figure 17. The Renishaw laser interferometer (RLU10) and previously calibrated capacitive displacement sensor were aligned along the same measurement axis to measure the movement of the target block. In other words, the output of the interferometer served as the reference value to calibrate the capacitive displacement sensor. The operation was also repeated 5 times.

**Figure 18:**
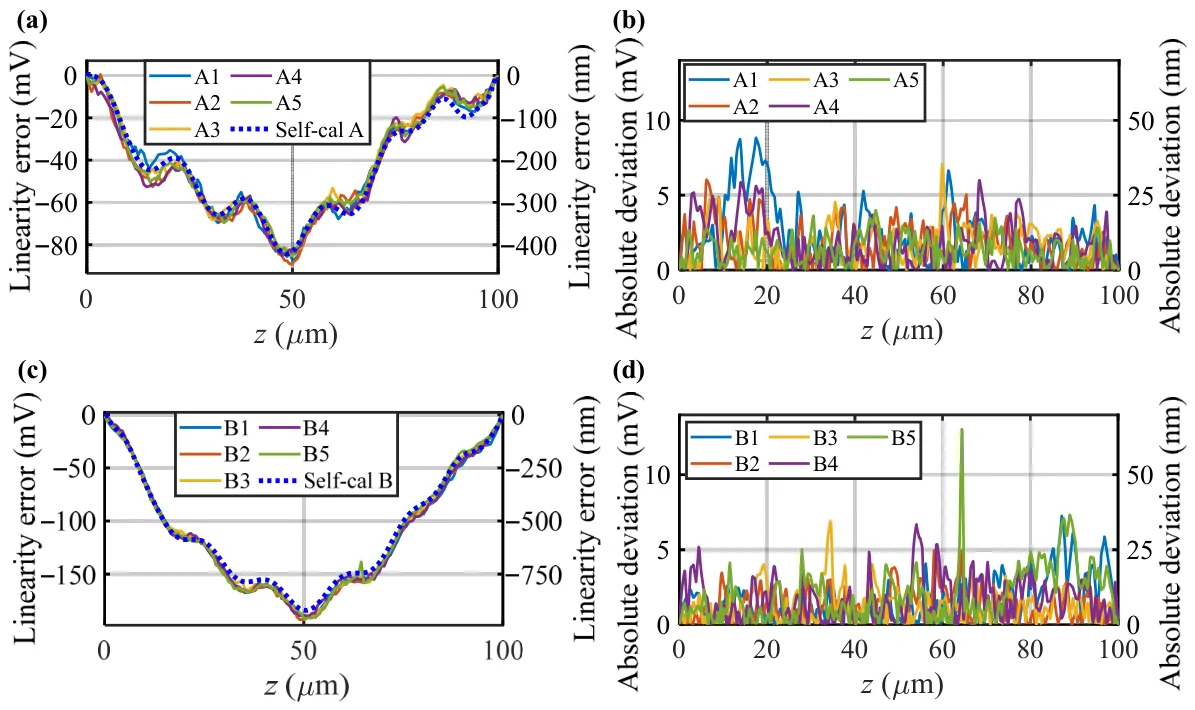
Verification of the self-calibration results by five repeated interferometer calibrations. (**a**) Verification linearity error result of Sensor A. (**b**) Verification repeatability of Sensor A. (**c**) Verification linearity error result of Sensor B (**d**). Verification repeatability of Sensor B.

Figure 18a,c show the linearity error curves of Sensors A and B, respectively, obtained using a laser interferometer as the reference. For comparison, the previously obtained self-calibration results were averaged and plotted in the same figures. Figure 18b,d show the differences between each of the five interferometer-based calibration results and their mean curves for Sensors A and B, respectively. Most values are within approximately ±25 nm, and no large monotonic discrepancy is observed across the range. The results demonstrate that the self-calibration method and the constructed setup can provide reliable calibration results without a higher-accuracy reference.

**Figure 19:**
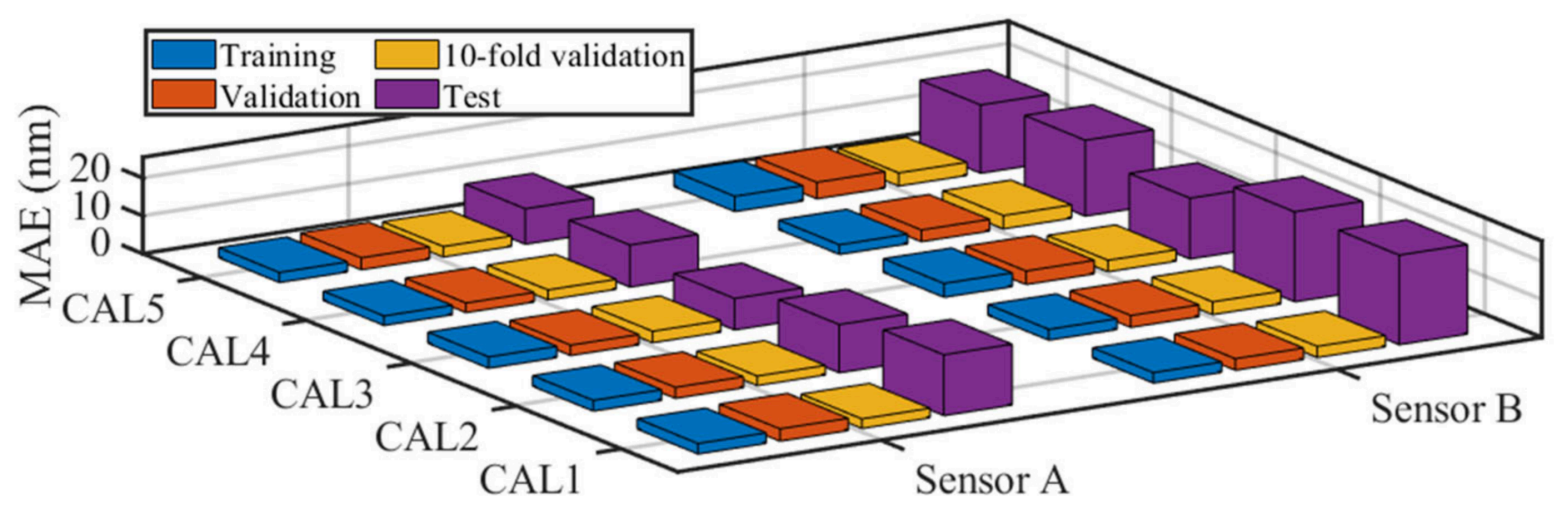
ANN performance.

The data from the five repeated self-calibration runs were finally reprocessed with ANN fitting. The same neural-network fitting and validation methods in Section 3.5 were used. The ANN’s training and validation performance were shown in Figure 19. The training, validation, and 10-fold cross-validation MAEs remained small and close to each other for all five calibration experiments and both sensors, indicating no evident overfitting. Unlike the simulation, in which the test error was calculated against a known theoretical true curve at unused positions, the true linearity-error curve was unavailable in the experiment. Therefore, the experimental test MAE was calculated from the difference between each ANN-based calibration result and the mean linearity-error curve obtained from five interferometer calibration results. Consequently, the test MAE includes not only the ANN fitting error but also the differences between the calibration and interferometer measurements and is therefore larger than the training and validation errors.

**Figure 20:**
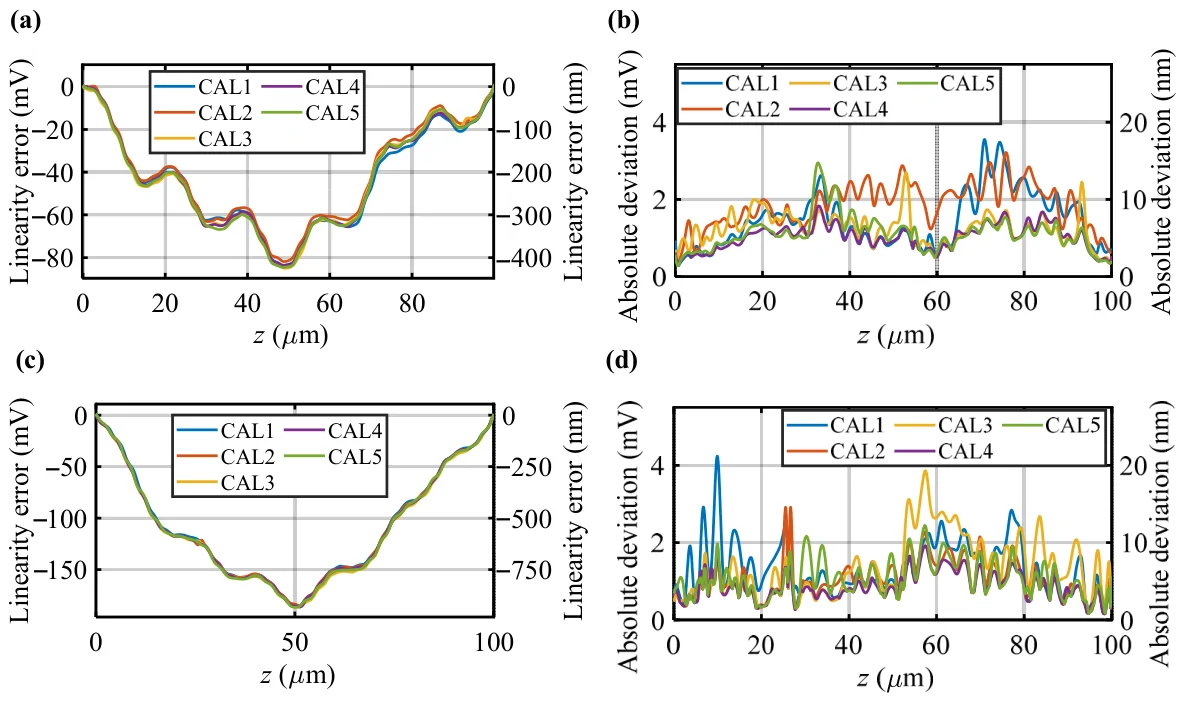
Five repeated self-calibration results using ANN fitting. (**a**) Calibration result of Sensor A. (**b**) Deviation curve of Sensor A’s calibration result. (**c**) Calibration result of Sensor B. (**d**) Deviation curve of Sensor B’s calibration result.

Figure 20a,c show five repeated self-calibration results obtained using ANN fitting. Figure 20b,d give the absolute deviation from the mean among the five self-calibration results.

**Figure 21:**
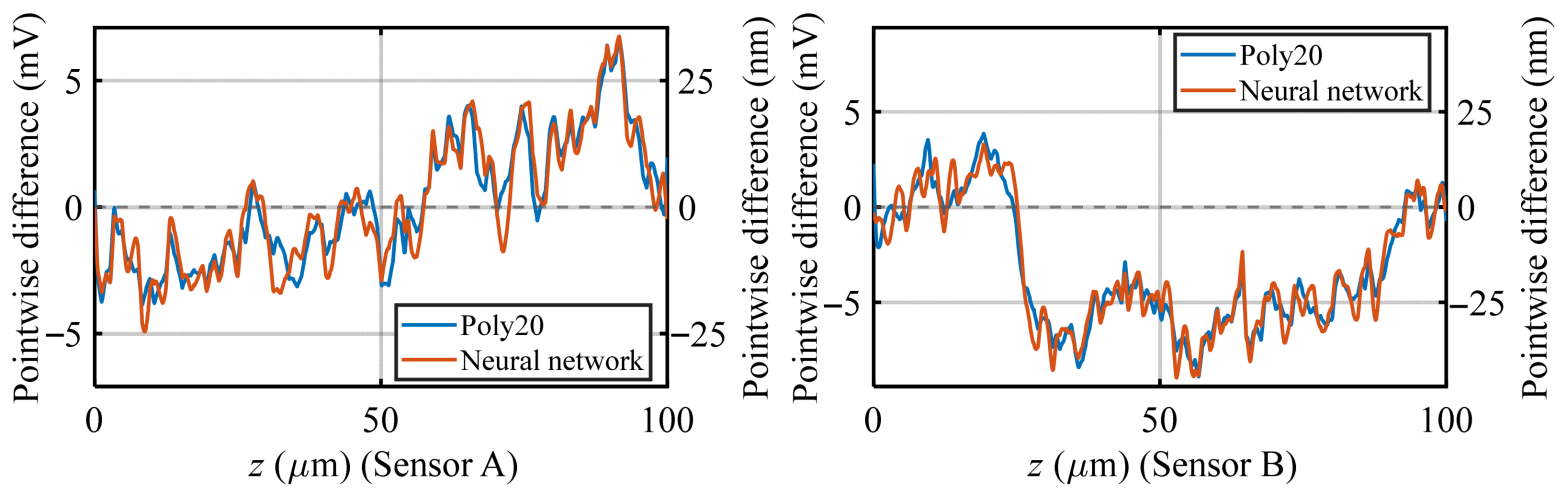
Pointwise differences between the mean calibration curves.

**Figure 22:**
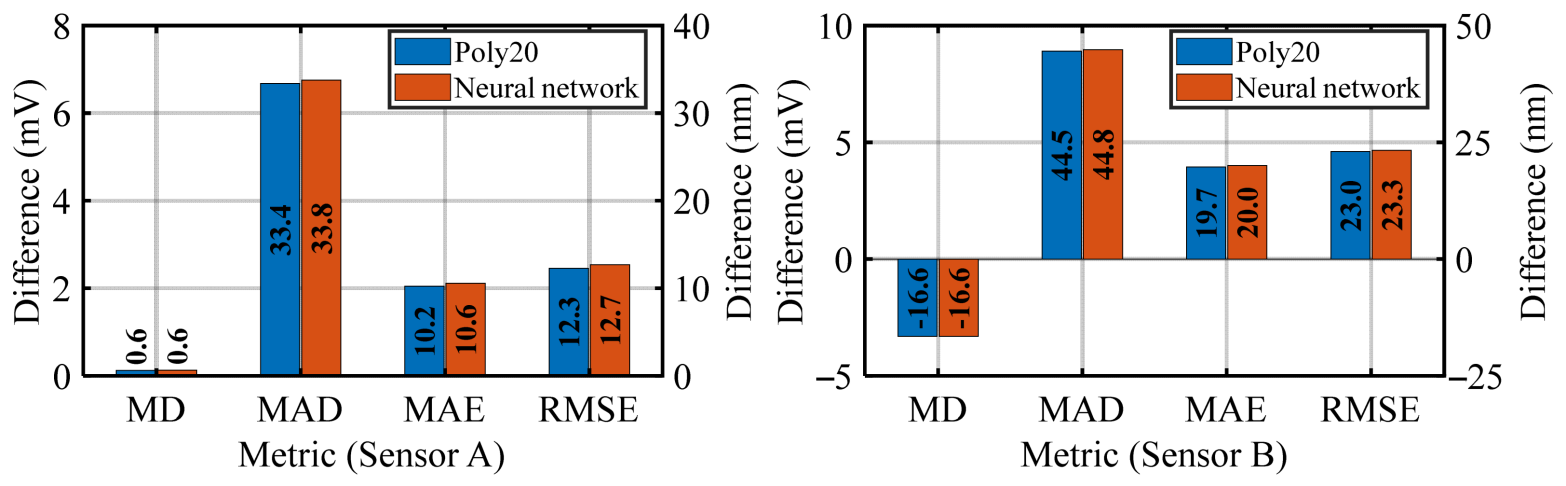
Differences between the mean self-calibration and interferometer-based calibration results.

To quantitatively evaluate the agreement between the two methods, for each sensor, the mean linearity-error curves from five polynomial-based and five ANN-based calibrations were compared with the mean curve from five interferometer-based calibrations on a common displacement grid. The signed pointwise difference was defined as the interferometer mean minus the corresponding self-calibration mean. Mean deviation (MD) is the mean value of these signed differences and describes the average offset between the curves. Maximum absolute deviation (MAD) is the largest absolute pointwise difference over the evaluated range. Mean absolute error (MAE) is the mean value of the absolute differences and describes the average discrepancy without cancellation between positive and negative values. Root mean square error (RMSE) is the square root of the mean squared differences and gives greater weight to larger discrepancies. The pointwise differences are shown in Figure 21, and the corresponding agreement metrics are presented in Figure 22, with numerical values listed in Table 2.

The experimental results show that self-calibration using either least-squares polynomial or ANN fitting recovers the main features of the interferometer-based linearity error curves for both sensors. The pointwise differences and the MD, MAD, MAE, and RMSE quantify the remaining disagreement between the mean curves. Across five repeated experiments, the self-calibration results exhibit deviations from their mean of approximately 15 nm or less, compared with approximately 25 nm for the interferometer-based results, indicating better repeatability under the present experimental conditions. The similar ANN training and validation errors, together with the 10-fold cross-validation results, provide no evident indication of overfitting to the calibration data. However, the variability of the interferometer-based measurements limits the experimental assessment of fine features in the linearity error curves. The present experiments therefore do not establish an accuracy advantage of ANN fitting over least-squares polynomial fitting or confirm its ability to recover actual high-frequency sensor nonlinearity. These results support the feasibility and repeatability of the proposed linearity-error self-calibration method, with independently determined sensitivity coefficients, while the differences from the interferometer-based results should be interpreted as agreement metrics rather than absolute calibration errors.

### 4.4. Monte Carlo Uncertainty Evaluation of the Self-Calibration Results

The MAE and maximum absolute deviation reported above quantify the agreement between the self-calibration and interferometric results. To further evaluate the uncertainty of the self-calibration results, a Monte Carlo method was applied in accordance with JCGM 101:2008 [51]. The considered factors were sensor noise, uncertainty in the sensitivity coefficients, uncertainty in the magnification ratios, and variation caused by ANN random initialization.

One experimental dataset containing 500 calibration points was used as the fixed input. All measured quantities were assumed to follow normal distributions. Independent noise was added to each voltage sample, using standard deviations of 0.043 mV for Sensor A and 0.038 mV for Sensor B. In each trial, one sensitivity coefficient and one magnification ratio were generated for each sensor and retained throughout the complete five-round calibration. The measured sensitivity coefficients were *k*_A_ = 0.19424 and *k*_B_ = 0.18490 V/μm, with standard deviations of 8.31158 × 10^−5^ and 1.73537 × 10^−4^ V/μm, respectively. The mean magnification ratios were *n*_A_ = 3.40458 and *n*_B_ = 3.63664, with standard deviations of 0.001749475 and 0.001361217, respectively. All these standard deviations assigned to the sensor noise, sensitivity coefficients *k*, and magnification ratios *n* were determined from repeated experimental measurements.

The variation associated with ANN fitting was included by assigning a different initialization seed to each ANN fitting operation. The network structure, training settings, and training–validation division were kept unchanged, and all seeds were recorded. In every trial, the voltage noise, sensitivity coefficients, magnification ratios, and ANN initialization seeds were varied simultaneously. Therefore, the resulting combined standard uncertainty can be expressed as

*u_c_* = *u_c_*(*V*, *k*, *n*, *ANN initialization*). (39)

**Figure 23:**
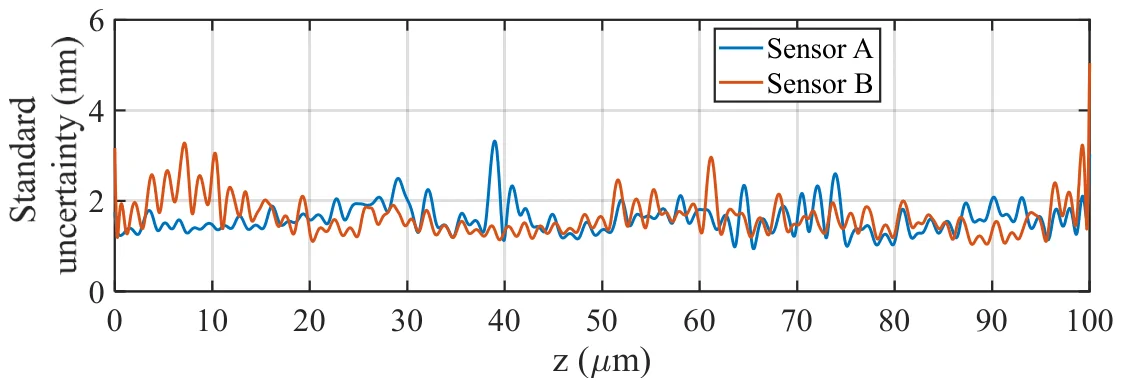
Standard uncertainty over the full range.

The complete five-round self-calibration was repeated *M* = 1000 times. The resulting linearity-error curves were evaluated on a common 0–100 μm grid, and the pointwise combined standard uncertainty was calculated as the standard deviation of the *M* results at each position. The empirical 95% coverage interval was obtained from the 2.5th and 97.5th percentiles. The convergence of the maximum pointwise combined standard uncertainty was also examined as *M* increased. The standard uncertainty at each position across the entire measurement range is shown in Figure 23.

**Figure 24:**
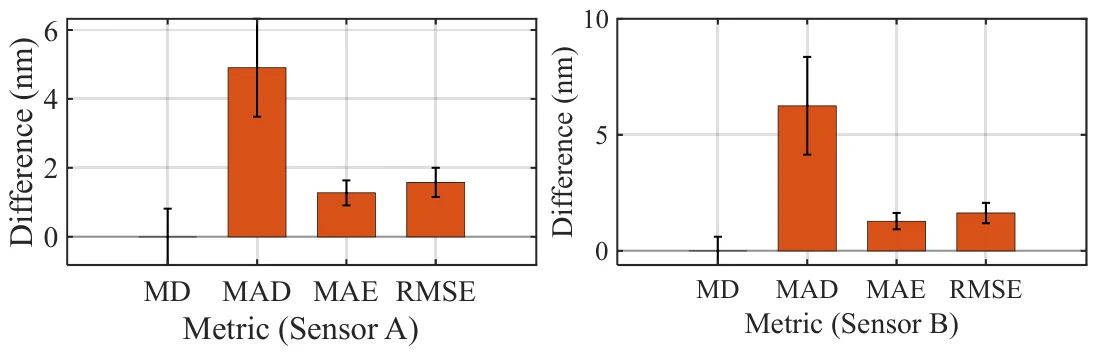
Deviation metrics of 1000 Monte Carlo trials.

Figure 24 shows the deviation metrics of the 1000 Monte Carlo calibration curves relative to their ensemble mean. The bars and error bars represent the mean and standard deviation of each metric across the 1000 trials, respectively. The MD remained close to zero, while the MAD was approximately 4.8 nm for Sensor A and 6.2 nm for Sensor B. The MAE and RMSE of the standard uncertainty remained below 2 nm for both sensors.

**Figure 25:**
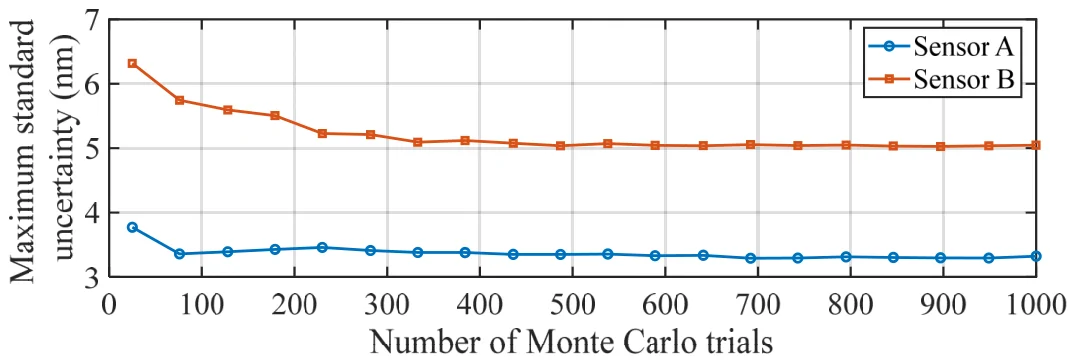
Convergence of standard uncertainty.

Figure 25 shows the convergence of the maximum pointwise combined standard uncertainty over the entire calibration range with increasing Monte Carlo trials. The maximum values over the entire range stabilized at approximately 3.3 nm for Sensor A and 5.0 nm for Sensor B after about 500 trials, indicating that 1000 trials were sufficient for the present uncertainty evaluation.

## 5. Discussion

The method is based on the calibration functions of the sensors and is therefore not limited only to capacitive displacement sensors. It can, in principle, be applied to other displacement-sensor technologies if the sensor output is continuous, repeatable with small linearity error over the calibration range. From a mathematical viewpoint, the linearity error cannot be specified only by its amplitude. The calibration function must remain monotonic, requiring

(40) $$\frac{dv_{R}\left(z\right)}{\mathrm{dz}}=k_{R}+\frac{de_{R}\left(z\right)}{\mathrm{dz}}>0,R=A,B.$$

In addition, the sensitivity coefficients must be known because the present method calibrates only the linearity error components *e*_A_(*z*) and *e*_B_(*z*), rather than the absolute displacement scale. In practical applications, if the sensitivity coefficient of one sensor is reliable, that of the other sensor can be determined from their output ratio.

The applicable range is limited by the sensor ranges and the dimensions of the rotating target. For the same allowable rotation angle, a longer lever provides a larger displacement range. For sensors sensitive to target inclination, the rotating lever can be replaced by a parallelogram-guided mechanism to maintain the target surface parallel to its initial orientation.

Once the sensitivity coefficients are known, the linearity-error calibration doesn’t require a higher-accuracy reference sensor. It can therefore be performed on site without routinely sending the sensors to an accredited calibration laboratory, reducing equipment cost, calibration service cost, and downtime.

Conventional traceable calibration is generally performed in a metrology laboratory with a controlled temperature of 20 °C, whereas the actual operating conditions may differ. Because the sensor linearity error can vary with the environment, a calibration made in a metrology laboratory may not fully represent the sensor behavior in use. The proposed method allows the linearity error to be evaluated directly under the actual operating conditions, provided that these conditions remain stable during calibration.

## 6. Conclusions

This study established the mathematical fundamentals of a self-calibration method in which two displacement sensors are mutually calibrated without using a higher-accuracy reference standard. The mathematical analysis showed that the final calibration error consists of two residual terms. The first term decreases as the number of calibration rounds increases and converges toward zero. In contrast, the fitting-error components generated in each calibration round do not converge to zero. Therefore, after the self-calibration process has converged, the fitting residual becomes the main source of the final calibration error.

The influences of the main factors were then investigated by simulation. For the sensor linearity error curves used in this study, the calibration results stabilized within five rounds, and approximately 500 sampling points provided stable fitting accuracy. Changes and mismatches in the magnification ratio from 2.5 to 4.5 caused less than 1 nm variation in MAE. A sensitivity mismatch of ±1% increased the MAE by several nanometers but did not prevent convergence. In comparison, thermal drift had a much greater influence. A drift of 240 nm increased the MAE to approximately 29 nm for Sensor A and 34 nm for Sensor B. These results indicate that reducing the calibration time and controlling the thermal drift are important for high-accuracy self-calibration.

The mathematical analysis also showed that repeated range segmentation increases the spatial-frequency content of the discrete calibration data. Compared with the 20th-order polynomial, the three-hidden-layer ANN followed the local high-frequency features more closely and reduced the fitting residual. In the simulation, the final-round MAEs by conventional least-squares polynomial fitting method were 6.960 nm and 5.155 nm for Sensor A and Sensor B respectively. The corresponding MAEs were reduced to 0.943 nm and 2.266 nm for Sensors A and B, respectively, by the ANN fitting method. It should be noted that the comparison was made between ANN and the conventional least-squares polynomial fitting method due to the reason that the latter had been used in the previous work of self-calibration [36]. Further comparison between ANN and other advanced fitting methods such as smoothing splines, regularized polynomial fitting will be made in future work. Spatial correlation between neighboring sampling points was not considered in this study and will be investigated in future work.

Based on the simulation results, an automated self-calibration setup was constructed. The calibration sampling time was reduced from approximately 1 h to 15 min. Five repeated self-calibration experiments showed that most absolute deviations from the mean were below 2 mV, and the maximum local deviation was approximately 3 mV, corresponding to about 15 nm. The self-calibration results were further verified using a Renishaw laser interferometer. The ANN-fitted experimental results had curve shapes and ranges consistent with the interferometer-based results. Their maximum absolute deviation from the mean was approximately 15 nm, compared with approximately 25 nm for the interferometer-based results. These results demonstrate that the proposed method can provide repeatable and reliable calibration of sensor linearity errors without using a higher-accuracy reference standard, provided that thermal drift and fitting residuals are properly controlled.

A preliminary Monte Carlo uncertainty evaluation included sensor noise, uncertainties in the sensitivity coefficients and magnification ratios, and variation caused by ANN initialization. The maximum pointwise combined standard uncertainties were approximately 3.3 nm for Sensor A and 5.0 nm for Sensor B for the considered factors. The experimental results support the feasibility and repeatability of the proposed method for calibrating sensor linearity errors with independently determined sensitivity coefficients.

## Figures and Tables

**Table 1:** Summary of influencing factors derived from the analysis and simulations.

Factor	Reason for Analysis	Result in This Study	Design Implication
Self-calibration round number	Controls attenuation of the first term in Equations (24) and (25)	The present curves stabilize by five rounds	Use five rounds as sufficient calibration rounds in this research; do not treat this as a universal value
Number of sampling points	Absent from the continuous equations but present in discrete fitting	Little additional benefit beyond about 500 points	Use 500 points as a practical value
Magnification ratio	Varies with installation and role exchange	Sub-nanometer change over the tested range	Maintain repeatability, but it is not the leading bottleneck
Sensitivity	Assumed known in the derivation	Moderate and bounded degradation for ±1% mismatch	Measure or compensate sensitivity before high-accuracy use
Thermal drift	Correlated with acquisition time and not removed by averaging	Approximately 29–34 nm MAE in the 240 nm drift scenario	Shorten acquisition and stabilize or estimate drift
Fitting residual	Non-vanishing term generated in every round	Polynomial: about 5–7 nm; neural network: about 0.9–2.3 nm in simulation	Use an ANN which is capable of fitting local high-frequency structure

**Table 2:** Differences between the mean self-calibration and interferometer-based calibration results.

Sensor	Fitting Method	MD (nm)	MAD (nm)	MAD (nm)	RMSE (nm)
A	Poly20	0.628	33.399	10.230	12.282
A	ANN	0.636	33.778	10.559	12.701
B	Poly20	−16.565	44.541	19.722	23.041
B	ANN	−16.560	44.840	20.014	23.286

## Data Availability

The data presented in this study are available on request from the corresponding author.
